# Self-Organization of Plant Vascular Systems: Claims and Counter-Claims about the Flux-Based Auxin Transport Model

**DOI:** 10.1371/journal.pone.0118238

**Published:** 2015-03-03

**Authors:** Chrystel Feller, Etienne Farcot, Christian Mazza

**Affiliations:** 1 Department of Mathematics and Swiss Institute of Bioinformatics, University of Fribourg, Fribourg, Switzerland; 2 School of Mathematical Sciences, University of Nottingham, Nottingham, UK; 3 Department of Mathematics and Swiss Institute of Bioinformatics, University of Fribourg, Fribourg, Switzerland; Instituto de Biología Molecular y Celular de Plantas, SPAIN

## Abstract

The plant hormone auxin plays a central role in growth and morphogenesis. In shoot apical meristems, auxin flux is polarized through its interplay with PIN proteins. Concentration-based mathematical models of the flux can explain some aspects of phyllotaxis for the L1 surface layer, where auxin accumulation points act as sinks and develop into primordia. The picture differs in the interior of the meristem, where the primordia act as auxin sources, leading to the initiation of the vascular system. Self-organization of the auxin flux involves large numbers of molecules and is difficult to treat by intuitive reasoning alone; mathematical models are therefore vital to understand these phenomena. We consider a leading computational model based on the so-called flux hypothesis. This model has been criticized and extended in various ways. One of the basic counter-arguments is that simulations yield auxin concentrations inside canals that are lower than those seen experimentally. Contrary to what is claimed in the literature, we show that the model can lead to higher concentrations within canals for significant parameter regimes. We then study the model in the usual case where the response function Φ defining the model is quadratic and unbounded, and show that the steady state vascular patterns are formed of loopless directed trees. Moreover, we show that PIN concentrations can diverge in finite time, thus explaining why previous simulation studies introduced cut-offs which force the system to have bounded PIN concentrations. Hence, contrary to previous claims, extreme PIN concentrations are not due to numerical problems but are intrinsic to the model. On the other hand, we show that PIN concentrations remain bounded for bounded Φ, and simulations show that in this case, loops can emerge at steady state.

## Introduction

Among its many functions, the plant hormone auxin is known to induce the formation of primordia in the shoot apical meristem, at locations where it accumulates at sufficient levels [1]. As these primordia become plant organs, the distribution of auxin at the microscopic scale of meristems is a fundamental determinant of the final shape of the plant at the macroscopic scale. The molecular mechanisms underlying auxin patterning are not fully known, but it is clearly established that auxin is actively transported between cells by transporter proteins, including the family of PIN proteins. Although it is known that the distribution of these transporters on cell membranes is asymmetric and depends on the distribution of auxin in neighbouring cells, the molecular details of this dependency remains a crucial unknown in the biology of auxin.

**Fig 1:**
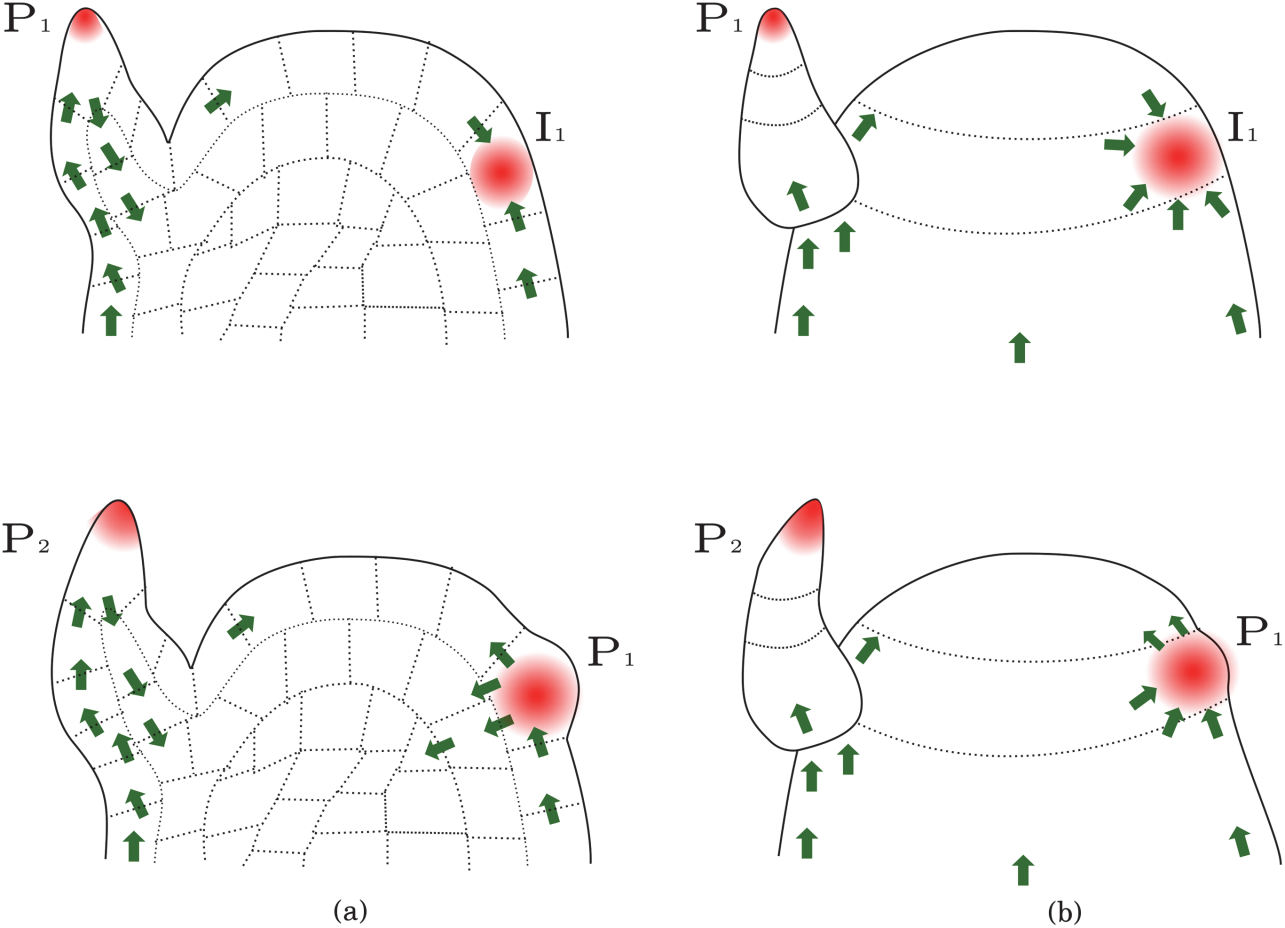
Schematic representation of a shoot apical meristem showing auxin canalization. (a) Transversal section. The upper panel shows PIN polarization (green arrows) soon after incipient primordium formation (*I*_1_), and the lower panel shows a later stage. (b) External view of the same meristem and developmental stages. PIN proteins are initially polarized towards auxin maxima close to primordia (upper panel). Notice that PIN polarization is later reversed in adaxial cells (lower panel).

PIN proteins have been identified as efflux carriers (i.e., they transport auxin out of the cell to the intercellular space), and are either oriented towards or against the auxin gradient, depending on the developmental stage of the primordium. More precisely, PIN proteins initially orient themselves towards a precise region of the meristem, away from older primordia. Auxin accumulates at the PIN convergence point, and induces the formation of a new primordium; during this phase, PIN are oriented against the auxin gradient. Incipient primordia then act as auxin sinks and induce auxin depletion in surrounding cells. Soon after initiation, PIN orientation switches in adaxial cells (i.e. cells placed on the side towards the axis of a primordium, usually the upper side) from “towards the center” to “towards the outside” (see Fig. 1), whereas PIN in the primordium orient themselves basally towards the inner tissue of the meristem (see Fig. 1 a). These processes lead to canalization, that is, to the development of vascular strands which transport auxin downward from the primordium to inner tissues. As reviewed by [2], PIN polarization reversal is enhanced by the increase of auxin synthesis in incipient primordia soon after their initiation [3, 4]. To date, it is not established how PIN is oriented in response to auxin gradients, see e.g. [5] for a recent review.

**Fig 2:**
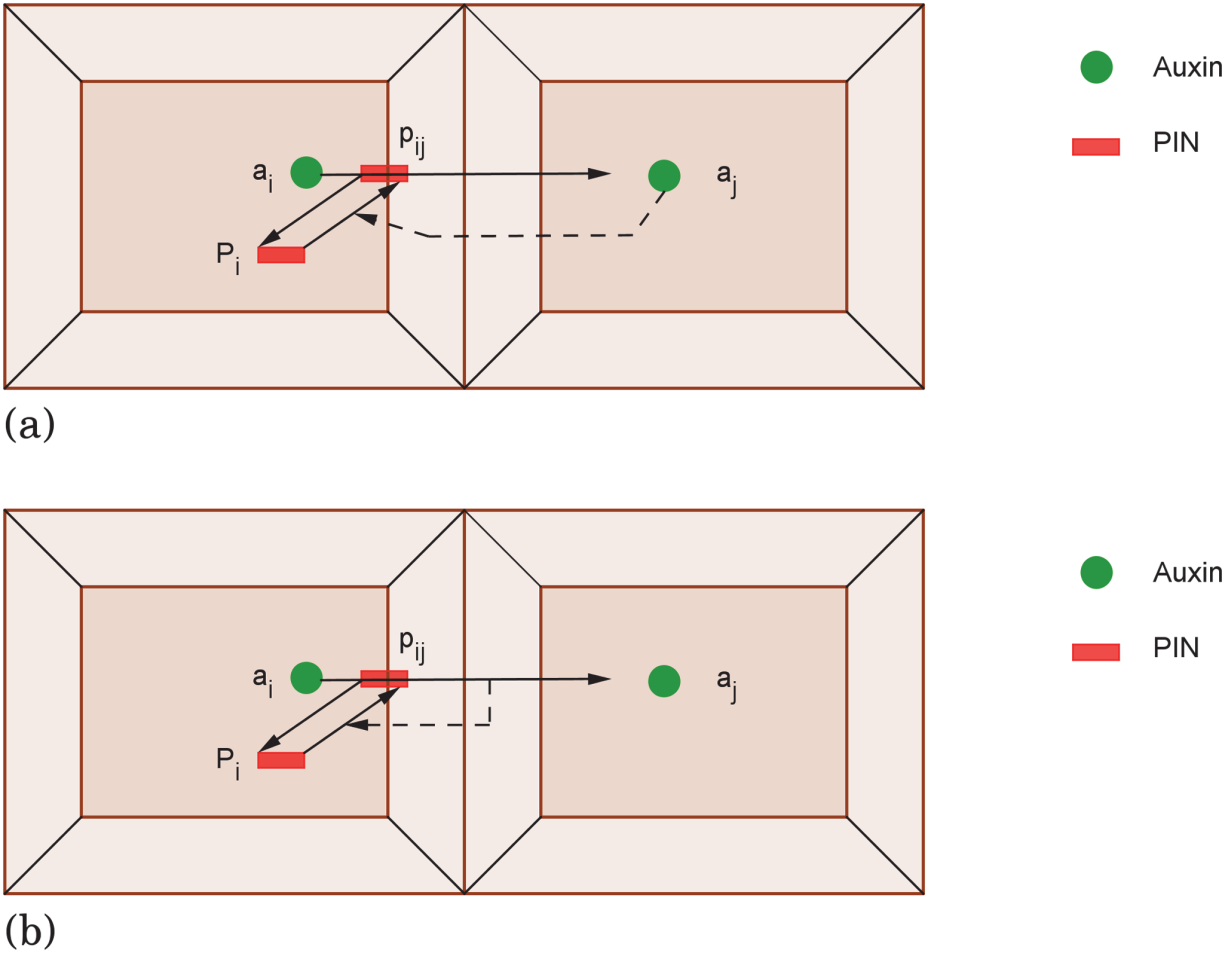
Schematic representation of the two different hypotheses for PIN orientation on membranes. (a) The concentration-based hypothesis: the PIN allocation to membrane *ij* is enhanced by the auxin concentration in the neighbouring cell *j*. (b) PIN concentration on membrane *ij* is increased according to the strength of the auxin flux through it. This mechanism is called the flux-based hypothesis.

The gaps in knowledge and the complexity induced by the large numbers of molecules at both intra- and inter-cellular scales make it difficult to treat the process by intuitive reasoning alone; mathematical models are therefore required to understand these phenomena and propose biologically plausible scenarios [6]. Based on experimental results, many authors including [7, 8, 9, 10, 11, 12] have built computational models relying on the following hypotheses: auxin is synthesized in all cells of the shoot apical meristem at rates that may vary, and is transported from cell to cell passively by diffusion and actively through efflux and influx carriers. The different models can be classified in two main classes, depending on the key hypothesis underlying PIN orientation (see Fig. 2):

- The **flux-based hypothesis** is based on the work of [13], and was first formalized by [14, 15] and later by [7, 10, 12, 16]. The assumption is that PIN concentration increases in membranes according to the strength of the auxin flux. This induces a positive feedback loop on the auxin flux, which is then amplified. It was first introduced as an intuitive explanation for the venation process. Models based on this hypothesis have been used to reproduce correctly the orientation of PIN proteins towards and against the auxin gradient [10], leading to the canalization/venation process. However, flux-based models have been criticized because of the lack of evidence for the existence of auxin flux sensors [17].
- The **concentration-based hypothesis** is based on the experimental result that PIN proteins orient towards incipient primordia, and thus presumably toward auxin maxima. In these models it is assumed that the rate at which PIN proteins accumulate on a membrane depends on the auxin concentration in the neighbouring cell [8, 9, 11]. How does a cell detect the auxin concentration in neighbouring cells? [9] cites an experimental result of [18] showing the negative feedback of auxin concentration on PIN endocytosis (a process which removes PIN from the membrane to the cytosol). Moreover, [19] proposed the following explanation: auxin accumulation in cells induces growth and expansion, which can be detected by the neighbouring cells due to mechanical forces applied on the cell membrane.

Most published studies have investigated models relying on one or both of the above hypotheses by means of computer simulations, aiming to reproduce realistic auxin distribution patterns in ‘virtual meristems’. However, any mathematical implementation of these hypotheses involves a large number of parameters, few of which are experimentally known to date. The exact patterning abilities of each model, sometimes the source of scientific controversy, thus cannot be fully characterized by these simulations: the fact that a particular type of pattern has not been observed in simulations might simply be explained by unexplored parameter regimes.

Until new experimental data more precisely explain the underlying molecular mechanisms and parameter values, the only possible definitive assessment of a model is through rigorous mathematical deduction and the derivation of formal proofs. As all existing models are high-dimensional and non-linear, only a few such results have been obtained to date.

In [11], concentration-based models have been shown to produce auxin patterns in a way that is similar to the well-known reaction-diffusion models, through transport-induced bifurcations from a steady state corresponding to a homogeneous distribution of auxin. In particular, these models can produce a variety of spotted and striped patterns, reminiscent of primordia and veins, respectively. The same class of models were investigated more recently in [20], where it was proved that typical patterns took the form of isolated blocks within a background of auxin-depleted cells. Using rigorous numerical bifurcation techniques, [21] were able to show that a typical bifurcation from a homogeneous auxin steady state could lead to either regularly-spaced spikes of auxin, or to stable oscillations. For flux-based models, [16] have shown that different distributions of PIN can lead to a homogeneous auxin distribution, and that these PIN patterns can be characterized in graph-theoretical terms. These authors also give conditions under which homogeneous auxin patterns are stable, and show that stable oscillations of auxin can occur in 1-D rows of cells through flux-based transport.

In this paper, we continue the study of flux-based models. We focus on the equations proposed by [10] as they have been able to reproduce PIN polarization toward incipient primordia (and thus against the auxin gradient), the reversed polarization (along the auxin gradient), phyllotactic patterns and venation. [10] proposed that the two different PIN polarization behaviours are consequences of different cellular responses to auxin. In the cells of the epidermal layer, the response function linking auxin flux to PIN insertion on the membrane is assumed to be linear, whereas the inner tissue response function is assumed quadratic.

However, several simulation studies raised problems for the model [22, 23], the canals were predicted to contain reduced auxin concentrations, while experimental studies show that auxin concentrations within canals can be higher than the background concentration, see, e.g., [24] or [25]. The model has thus been extended in various ways. [26] modeled auxin influx carriers. [23] combined both (the concentration- and flux-based hypotheses) in a hybrid model, each cell having both (flux- and concentration-based PIN cycling) depending on the auxin concentration. [12] modified the model of [10] by also considering PIN cycling and the concentration within the cytosol of each cell. They argued that it is then able to predict canals with higher auxin concentration than in the surrounding tissues.

Here we will show that the model of [10] is itself able to generate auxin canals and PIN polarization with and against auxin gradients, and prove that it is in fact able to create canals having higher auxin concentrations than those in the surrounding cells. Mathematical proofs are given that lead to precise parameter regimes that yield such behaviours. Hence, contrary to what is claimed in the literature, the model reproduces patterns of auxin concentrations like those seen in nature. Previous studies dealing with an unbounded response function introduced a cut-off [12] or halted simulations [7] when auxin and PIN concentrations became too high; the authors argued that this behaviour was due to numerical problems. We prove that an intrinsic property of the model is divergence in finite time, and thus that high values of PIN concentration are not due to numerical problems but are a consequence of the computational model itself. We finally study the steady state patterns. These are, at least for quadratic response functions, composed of directed forests of directed trees rooted at sinks, in the absence of cycles. They thus ressemble the plant vascular system. Special emphasis is given to source- and sink-driven systems, for which exact solutions are provided.

## Results

### The flux model

**Fig 3:**
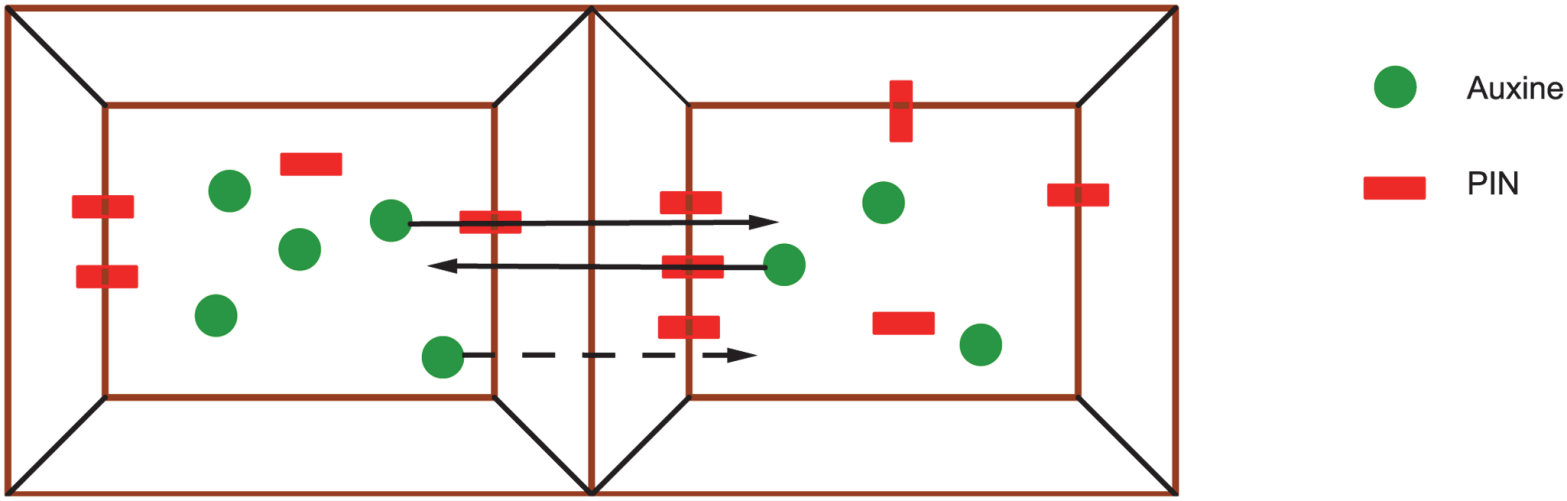
Localisation of auxin molecules and PIN proteins in two neighbouring cells.

We suppose that the set of cells *i* ∈ *V* = {1, ⋯, *M*} is arranged as a graph 𝓖=(V,E). The edge set is composed of pairs of cells (*i*, *j*) which are nearest neighbours, denoted as *i* ∼ *j* in what follows. The flux model describes the time evolution of the concentrations of auxin molecules *a*_*i*_(*t*) and of PIN proteins *p*_*i*_(*t*) within each cell *i*. We thus need the following variables, for each cell *i* and for each interface (*i* → *j*), where some of the PIN proteins which are contained in cell *i* are positioned, facing cell *j*, see Fig. 3:

$$\begin{array}{ccc} a_{i} & = & \text{auxin}\;\text{concentration}\;\text{in}\;\text{cell}\;i\;\text{in}\;\text{mol}\;m^{-3}\text{.}\\ p_{i} & = & \text{PIN}\;\text{concentration}\;\text{in}\;\text{cell}\;i\;\text{in}\;\text{mol}\;m^{-3}\text{.}\\ p_{ij} & = & \text{PIN}\;\text{concentration}\;\text{on}\;\text{the}\;\text{membrane}\;\text{of}\;\text{cell}\;i\;\text{facing}\;\text{cell}\;j\;\text{in}\;\text{mol}\;m^{-2}\text{.}\\ V_{i} & = & \text{volume}\;\text{of}\;\text{cell}\;i\;\text{in}\;m^{3}\text{.}\\ S_{ij} & = & \text{surface}\;\text{area}\;\text{of}\;\text{the}\;\text{membrane}\;\text{between}\;\text{cell}\;i\;\text{and}\;j\;\text{in}\;m^{2}\\ N_{i} & := & \text{set}\;\text{of}\;\text{neighbours}\;\text{of}\;\text{cell}\;i\text{.}\; \end{array}$$

All the parameters and variables used in this work are summarized in Table 1.

**Table 1:** Notations.

𝓖=(V,E)	Graph of node set *V* and of edge set *E*.
${𝓖}^{*}=(V^{*},E^{*})$	Oriented sub-graph of node set V* and of oriented edge set E*.
I* = *V*∖V*	Set of isolated cells of $\bar{𝓖}$.
*i* ∼ *j*	Neighbouring cells *i* and *j* with (*i*, *j*) ∈ *E*.
*M* = ∣*V*∣	Number of nodes of the graph 𝓖.
$N_{i}$	Neighbourhood of cell *i*, that is $N_{i}\;=\;\{k\;\in \;V\;\;|\;\;k\;\sim \;i\}$.
*d*_*i*_	Number of neighbours of cell *i*, i.e. $d_{i}=|N_{i}|$.
*m*	Number of membranes of the graph 𝓖, i.e. *m* = |*E*|.
*a*_*i*_	Auxin concentration in cell *i*.
*a*_*ij*_	Auxin concentration in the membrane of cell *i* facing cell *j*.
*P*_*i*_	PIN concentration in cell *i*.
*p*_*ij*_	PIN concentration on the membrane of cell *i* facing cell *j*.
*V*_*i*_	Volume of cell *i*.
*S*_*ij*_	Surface area of the membrane separating cell *i* from cell *j*.
*W*	Ratio of the surface area of membrane *ij* to the cell volume, when the cells are regular.
$J_{i\to j}^{D}$	Auxin flux through the membrane between cell *i* and *j* due the diffusion.
$J_{i\to j}^{A}$	Auxin flux through the membrane between cell *i* and *j* due the PIN active transport.
*J*_*i* → *j*_	$J_{i\to j}^{D}+J_{i\to j}^{A}=$ net flux through the membrane *ij*.
*γ*_*D*_	Auxin diffusion rate.
*γ*_*A*_	Auxin active transport rate.
*D*	Normalized auxin diffusion rate.
*T*	Normalized active transport rate.
$\alpha_{a}^{i}$	Auxin synthesis rate in cell *i*.
$\beta_{a}^{i}$	Auxin degradation rate in cell *i*.
$\alpha_{p}^{i}$	PIN production rate in cell *i*.
$\beta_{p}^{i}$	PIN degradation rate in cell *i*.
*κ*	Saturating constant.
*μ*	Background PIN removal rate from the membranes.
*λ*	PIN insertion rate in the membrane *ij*.
*h*	Non-linear response function.
Φ(*x*)	= *h*(*x*/*W*).

Following [10], we will consider the flux associated with diffusion and active transport.

$$\begin{array}{ccc} J_{i\to j}^{D} & = & \text{diffusive}\;\text{auxin}\;\text{flux}\;\text{through}\;\text{the}\;\text{membrane}\;\text{between}\;\text{cell}\;i\;\text{and}\;j\;\text{in}\;\text{mol}\;m^{-2}s^{-1}\text{,}\\ J_{i\to j}^{A} & = & \text{auxin}\;\text{flux}\;\text{associated}\;\text{with}\;\text{active}\;\text{transport,}\\ J_{i\to j} & = & J_{i\to j}^{D}+J_{i\to j}^{A}=\text{net}\;\text{flux}\;\text{through}\;\text{the}\;\text{membrane}\;ij. \end{array}$$

$J_{i\to j}^{D}$ is modelled using Fick’s law as $J_{i\to j}^{D}=\gamma_{D}(a_{i}-a_{j})$ where *γ*_*D*_ (*ms* ^−1^) is a permeability constant reflecting the capability of auxin to cross the membrane. The flux due to active transport across a membrane separating cells *i* and *j* is modelled as $J_{i\to j}^{A}=\gamma_{A}(a_{i}p_{ij}-a_{j}p_{ji})$, where *γ*_*A*_ (*m* ^3^mol^−1^ *s* ^−1^) characterizes the transport efficiency of the PIN pumps.

[10] proposed the following set of ordinary differential equations

$$\begin{array}{cc} \frac{da_{i}}{dt} & =\alpha_{a}^{i}-\beta_{a}^{i}a_{i}-\frac{1}{V_{i}}\sum_{j∼i}S_{ij}J_{i\to j}, \end{array}$$ (1a)

$$\begin{array}{cc} \frac{dp_{ij}}{dt} & =h\left(J_{i\to j}\right)+\rho_{0}-\mu p_{ij}, \end{array}$$ (1b)

where the coefficients $\alpha_{a}^{i}$ (mol *m* ^−3^ *s* ^−1^) and $\beta_{a}^{i}$ (*s* ^−1^) are auxin production and degradation factors of cell *i*. *ρ*_0_ (mol *m* ^−2^ *s* ^−1^) is the basal PIN insertion rate into the membrane, *μ* (*s* ^−1^) is the basal removal rate from the membrane. The first equation (1a) gives the rate of variation of auxin concentration in cell *i* as a function of protein production and degradation, and of the flux *J*_*i*→*j*_ through the membrane *ij*. The second equation reflects [13] original concept that canalization is induced by a positive feedback between flux and transport. This canalization hypothesis was then formalized in [14, 15]. According to the second equation (1b), the variation of the concentration *p*_*ij*_ of PIN proteins transporting auxin to cell *j* is a consequence of 1) insertion in the membrane induced by the flux and 2) basal insertion and removal of PIN protein from the membrane. The intensity of PIN insertion into the membrane *i* is modelled using a **non-linear response function** *h*. The positive feedback mechanism is modelled by assuming that *h* is increasing and non-negative. It is furthermore assumed that no PIN proteins are removed from the membrane when the number of incoming auxin molecules is larger than the number of outgoing auxin molecules; in mathematical terms, this means that

$$\begin{array}{c} h(J)=0\;\text{when}\;J\leq 0. \end{array}$$ (2)

Typical examples of such functions are

$$\begin{array}{c} \text{linear:}\;h_{L}\left(J_{i\to n}\right)≔\kappa \frac{J_{i\to n}}{J_{ref}},\;\;\text{or}\;\text{quadratic:}\;\;h_{Q}\left(J_{i\to n}\right)≔\kappa \;{\left(\frac{J_{i\to n}}{J_{ref}}\right)}^{2}, \end{array}$$

where *κ* is a positive parameter in mol *m* ^−2^ *s* ^−1^ and *J*_*ref*_ is an arbitrary reference flux in mol *m* ^−2^ *s* ^−1^. [7] also consider functions of the form

$$\begin{array}{c} h_{1}\left(J\right)=\rho \frac{J^{n}}{\theta^{n}+J^{n}},\;\;h_{2}\left(J\right)=\rho J^{n}. \end{array}$$

It is important to note that the properties of the steady states associated with the o.d.e. (1) strongly depend in general on the nature of *h* [7, 27]. We will provide more precise results on this topic in what follows. In [7, 12, 16] a modified model is also considered where PIN transport (between the membrane and the cytosol) is distinguished from PIN degradation/creation in each cell *i*. We follow the version proposed in [16] which includes the time evolution of the PIN concentrations *P*_*i*_(*t*) in the cytosol of each cell *i*, and takes the form:

$$\begin{array}{cc} \frac{da_{i}}{dt} & =\alpha_{a}^{i}-\beta_{a}^{i}a_{i}-\frac{1}{V_{i}}\sum_{j∼i}S_{i,j}\;\left(\gamma_{D}\left(a_{j}-a_{i}\right)+\gamma_{A}\left(a_{i}p_{ij}-a_{j}p_{ji}\right)\right), \end{array}$$ (3a)

$$\begin{array}{cc} \frac{dP_{i}}{dt} & =\alpha_{p}-\beta_{p}P_{i}+\frac{1}{V_{i}}\sum_{j∼i}S_{ij}\;\left(\mu p_{ij}-\lambda P_{i}h\left(J_{i\to j}\right)\right), \end{array}$$ (3b)

$$\begin{array}{cc} \frac{dp_{ij}}{dt} & =\lambda P_{i}h\left(J_{i\to j}\right)-\mu p_{ij},\;\text{when}\;i∼j, \end{array}$$ (3c)

where *μ* is the removal rate of PIN from membrane. For this new model, the global PIN concentration inside each cell is preserved when *α*_*p*_ = *β*_*p*_ = 0, that is,

$$\begin{array}{c} \frac{dP_{i}\left(t\right)}{dt}+\frac{1}{V_{i}}\sum_{j∼i}S_{ij}\frac{dP_{ij}\left(t\right)}{dt}\equiv 0. \end{array}$$

Let ***a*** = (*a*_*i*_)_*i* ∈ *V*_, ***P*** = (*P*_*i*_)_*i* ∈ *V*_ and ***P*** = (*p*_*ij*_)_(*i*, *j*) ∈ *E*_ be the vector containing auxin and PIN concentrations in the cells and on the membranes.

[10] studied the system of differential equation (1) numerically, letting vary parameter values including the auxin synthesis and degradation rates in each cell *i*. They were able to reproduce PIN polarization with and against auxin gradients. The system with *h*(*x*) = *x* ^2^ can also reproduce the canalization phenomenon where canals formed by PIN polarization are surrounded by cells without transporter. With a linear feedback function *h*(*x*) = *x*, [10] reproduced phyllotactic patterns and an inhibiting field.

[12] showed using simulations that [10]’s model is able to generate phyllotactic patterns and canalization without changing the feedback function *h*, simply by altering the auxin synthesis/degradation rates. Indeed, if incipient primordia acts as sinks by degrading auxin at higher rates than other cells, they attract and accumulate auxin molecules. The primordia then become sources as they grow, inducing the initiation of the inner plant vascular system.

From the fact that *h*(*x*) = 0 for *x* ≤ 0, the equilibrium (a*, P*, P*) requires that for every pair of nearest neighbour cells *i* ∼ *j*, either $p_{ij}^{*}=0$ or $p_{ji}^{*}=0$. This property allows an orientation of the graph 𝓖 to be associated with each steady state, where an edge (*i*, *j*) is oriented as *i* → *j* if and only if $p_{ij}^{*}>0$. Note that with this procedure *p*_*ij*_ = *p*_*ji*_ = 0 implies that the corresponding edge is actually removed in the oriented graph; the procedure thus leads to an oriented sub-graph denoted by $\bar{𝓖}$ in what follows. This oriented graph was defined in [16], where it was used to characterize further properties of steady states with homogeneous auxin, i.e. *a*_*i*_ = a* ∀*i* by the condition that $\bar{𝓖}$ must be well-balanced.

### Model simplification

Consider a graph 𝓖=(V,E) with regular cells, i.e., the surface areas between membranes *S*_*ij*_ and the cell volumes *V*_*i*_ are all equal. Let $W\equiv \frac{S_{ij}}{V_{i}},$ ∀(*i, j*) ∈ *E*. Set:

$$\begin{array}{c} D=\gamma_{D}W,\;T=\gamma_{A}W\;\text{and}\;\Phi \left(x\right)=h\;\left(\frac{x}{W}\right). \end{array}$$

We will focus on a simplified model which is obtained by assuming that

$$\begin{array}{c} \alpha_{p}=\frac{\alpha_{p}^{0}}{\varepsilon },\;\beta_{p}=\frac{\beta_{p}^{0}}{\varepsilon }\;\text{and}\;D=D^{0}\varepsilon , \end{array}$$

for a small parameter *ɛ* ≈ 0. This regime assumes that (passive) diffusion is negligible compared to active transport. Without loss of generality, we assume that *T* = 1. In this regime, $P_{i}=\frac{\alpha_{p}}{\beta_{p}}$ and (3) becomes

$$\begin{array}{c} \begin{array}{cc} \frac{da_{i}}{dt} & =\alpha_{a}^{i}-\beta_{a}^{i}a_{i}+\sum_{k∼i}\left(a_{k}p_{ki}-a_{i}p_{ik}\right),\;\text{for}\;j\in V\\ \frac{dp_{ij}}{dt} & =\lambda \frac{\alpha_{p}}{\beta_{p}}\Phi \left(J_{i\to j}\right)-\mu p_{ij},\;\forall i,j\in V,i∼j \end{array} \end{array}$$ (St0)

where *J*_*i* → *j*_ = (*a*_*i*_ *p*_*ij*_ − *a*_*j*_ *p*_*ji*_).

We show moreover in the Supporting Text that if a solution $\bar{\text{x}}(t)=(\bar{\text{a}}(t),\bar{\text{P}}(t))$ of (St0) converges towards an equilibrium x* = (a*, P*), then, for small enough *ɛ*, the solution (***a*** ^*ɛ*^(*t*), ***P*** ^*ɛ*^(*t*), ***P*** ^*ɛ*^(*t*)) of (3) having the same initial condition remains in a small neighbourhood of $\bar{\text{x}}(t)$ for all future time.

### Divergence in finite time

Most of the papers dealing with (1) or (St0) with unbounded Φ introduce a cut-off or stopped simulations when the values of PIN concentrations *p*_*ij*_ became too large (see, e.g., [7, 12]). These papers argue that this divergence is due to numerical problems. We show that these extreme values are not due to numerical problems, but that the *p*_*ij*_(*t*) can diverge toward ∞ in finite time; this is hence an intrinsic property of the computational model. We prove in S1 Text that the slow equation (St0) has an unique solution, which is defined for all *t* ≥ 0 when Φ is a bounded function if $\alpha_{a}^{i},\beta_{a}^{i},\mu ,\lambda ,\alpha_{p}/\beta_{p}>0$, ∀*i* ∈ *V*. When Φ is not bounded, either the solution is defined for all *t* ≥ 0, or only for *t* in a bounded interval [0, *δ*); in the latter case, the solution **diverges toward ∞** as *t* → *δ*. An example where PIN concentrations on membrane diverge in a finite time is provided in the supporting information.

### Steady state topologies

As discussed above, every steady state leads to an oriented sub-graph $\bar{𝓖}=(V,E^{*})$ of the basic graph 𝓖=(V,E). For every pair of nearest neighbours *i* ∼ *j* of *E*, E* either does not contain an oriented edge linking these two cells, or contains at most one of the two possible directed edges (*i* → *j*) and (*j* → *i*). Hence,

$$\begin{array}{c} \left(i\to j\right)\in E^{*}\;\text{if}\;\text{and}\;\text{only}\;\text{if}\;p_{ij}^{*}\neq 0=p_{ji}^{*}. \end{array}$$

Let I* be the set of all isolated cells of $\bar{𝓖}$ (i.e.those cells *i*_0_ for which *♯*{*k* ← *i*_0_} = 0 and *♯*{*k* → *i*_0_} = 0). For clarity, denote by ${𝓖}^{*}$ the sub-graph of $\bar{𝓖}$ where the isolated cells have been removed, i.e., ${𝓖}^{*}$ is the directed graph of edge set E*, and of node set V* = *V*\I*. Note that if $\beta_{a}^{i}=0$ for some cell *i*, then necessarily $a_{i}^{*}<+\infty$ implies that *i* ∉ I*.

**Fig 4:**
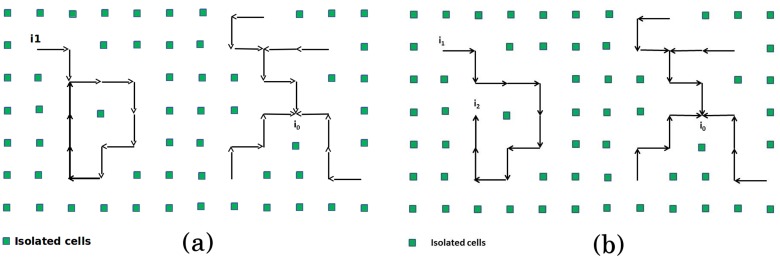
Examples of oriented sub-graph 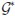. (a) A sub-graph 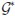 (in black) in a background of isolated cells. *i*_1_ is a source while *i*_0_ is a sink. (b) A directed forest 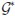 (in black) in a background of isolated cells. *i*_1_ is a source while *i*_0_ and *i*_2_ are sink cells.

Let ${𝓖}^{*}$ be an oriented sub-graph of 𝓖. The following notions will be useful in what follows, see Fig. 4 a):

- The **out-degree** (resp. **in-degree**) of a cell *i* in ${𝓖}^{*}$ is the number of cells *j* such that *i* → *j* (resp. *j* → *i*). The **degree** is the sum of the out-degree and the in-degree.
- A **sink** of ${𝓖}^{*}$ is a cell with out-degree 0 and in-degree > 0.
- A **source** of ${𝓖}^{*}$ is a cell with in-degree 0 and out-degree > 0.

### Stable steady state topologies

This section focuses on the stability of the system (St0) with a quadratic Φ(*x*) = *x* ^2^. Let $E_{0}=(\alpha_{a}^{1}/\beta_{a}^{1},\cdots ,\alpha_{a}^{M}/\beta_{a}^{M},0,\cdots \;0)$ be the configuration with every *p*_*ij*_ equal to zero, i.e., the associated directed graph is composed of isolated nodes only. For our choice of the function Φ, this configuration is always asymptotically stable, see [16]. From the latter, we also know that steady states with homogeneous auxin and non-zero PIN exist for well-balanced orientations ${𝓖}^{*}$, but their stability is unknown in general. We now prove that for a quadratic Φ, the stability of arbitrary steady states (i.e., where auxin is not necessarily homogeneous) can in fact be characterized using some simple necessary conditions on the sub-graph ${𝓖}^{*}$.

#### Instability criterion

**Theorem 1** *Consider the system* (St0) *with* Φ(*x*) = *x* ^2^. *Assume that* $\alpha_{a}^{i}>0$, $\beta_{a}^{i}\geq 0$, ∀*i* ∈ *V*, *and that* *μ*, *c* > 0. *Let* (a*, P*) ≠ *E*_0_ *be a bounded equilibrium of the system (St0) of associated oriented sub-graph* ${𝓖}^{*}$. *Then*,

- *If* ${𝓖}^{*}$ *contains no sink cells, then* (a*, P*) *is unstable*.
- *If* (a*, P*) *is stable, then* ${𝓖}^{*}$ *is an oriented sub-graph of G composed of trees directed from leaves to roots such that every cell i* ∈ *V has at out-degree* ≤ *1*.

We hence observe that every stable equilibrium is obtained by considering an oriented graph ${𝓖}^{*}$ **composed of directed trees pointing to sinks**, see Fig. 4 b). The stable patterns do not contain loops, as it has been observed through simulations in most of the papers dealing with the flux model. Simulations show that loops can obtained by using a bounded function Φ.

**Fig 5:**
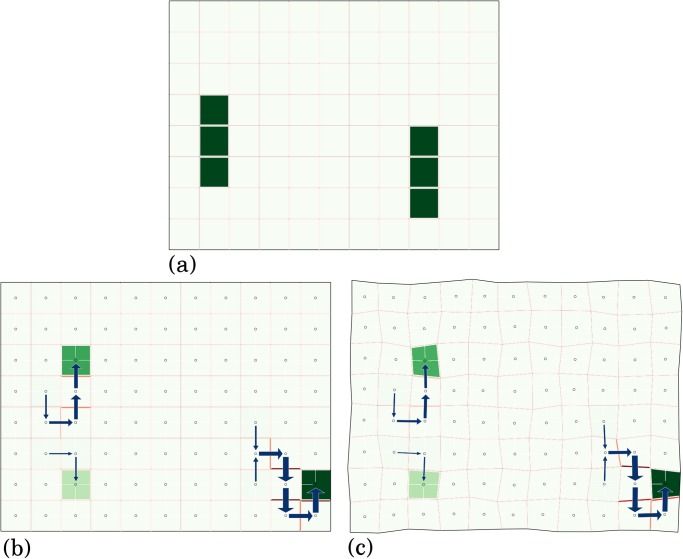
Steady state equilibrium in a simulation on a regular grid of cells with heterogeneous auxin production rates. (a) Initial conditions using a grid of 88 cells where most cells have an auxin production rate $\alpha_{a}^{i}=0.1$ except the two groups of 3 cells shown in darker green, where $\alpha_{a}^{i}=2.1$. (b) Using the production rates from (a) and parameters $\left(\beta_{a}^{i},\lambda ,\mu )=(5,5,0.1\right)$ in every cell, one of the possible steady state solutions. Each cell is colored in green according to the auxin concentration *a*_*i*_ (pale for the minimal concentration, dark green for the maximal). Cell membranes are colored according to the corresponding PIN concentration (with a red colormap). The flux *J*_*i* → *j*_ is represented by a blue arrow of width proportional to ∣*J*_*i* → *j*_∣. (c) With parameters and initial conditions identical to those in (b) but with a diffusion coefficient *D* = 0.001 and a grid of non-regular cells.

To obtain further confirmation of Theorem 1, we performed numerical simulations on a regular grid of cells. In most simulations with homogeneous auxin production rates $\alpha_{a}^{i}=\alpha_{a}\;\forall i$, the simulations ended on the steady-state *E*_0_, where all cells are isolated. We thus considered a grid of 8 × 11 cells within which two groups of three cells have a higher production rate, see Fig. 5 a) and b). We observe that simulation seems to converge to a state composed of rooted trees as predicted by Theorem 1. The S1 Text provides also precise formulas for computing locally asymptotically stable configurations for quadratic response functions.

Note that the result above applies at a limit where the diffusion constant vanishes *D* = 0, and for regular tissues where all cells have the same volume and surface area. Neither of these two conditions is likely to be strictly satisfied in real systems, but they greatly simplify mathematical analysis. Also, we expect Theorem 1 to remain true for systems where the two conditions are nearly satisfied, i.e. where *D* is non-zero but small, and where cells do not vary too much in size. This intuition was confirmed by numerical simulations, as seen in Fig. 5 c) where the previously observed steady-state is qualitatively unchanged by slightly relaxing the two conditions.

We next focus on **source and sink driven systems**, which play a fundamental role in plant growth, see, e.g., [12].

### Sink-driven system

As explained in the Introduction, experiments show that the primordia of the L1 layer act first as auxin sinks, inducing auxin depletion in surrounding cells. We study the stable patterns associated with this phase within the flux-based modelling framework, in the extreme case where there is a single primordium, which is located at some site *i*_0_ such that

$$\begin{array}{c} \beta_{a}^{i}\approx 0,\;\forall i\neq i_{0}\;\text{and}\;\beta_{a}^{i_{0}}>0. \end{array}$$

**Fig 6:**
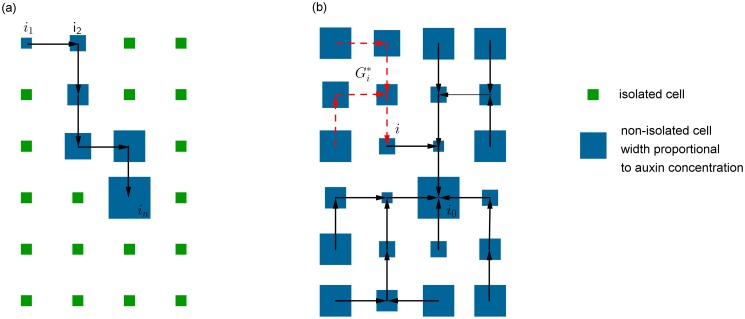
Schema illustrating source- and sink-driven vascular patterns when there is a single primordium *i*_0_. The sizes of the blue boxes represent auxin concentrations. In the left panel, the source *i*_0_ creates a linear vein within which the auxin concentration increases; auxin concentrations within the vein are higher than the background auxin level (A). In the right panel, the patterning process leads to a directed spanning tree rooted at the unique sink *i*_0_. The auxin concentration *a*_*i*_ inside each cell *i* is proportional to the inverse of the size of the sub-tree 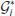 of 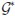 rooted at *i*. Auxin concentrations thus decrease along the paths, leading to an auxin depleted zone in the neighbourhood of the sink *i*_0_.

**Fig 7:**
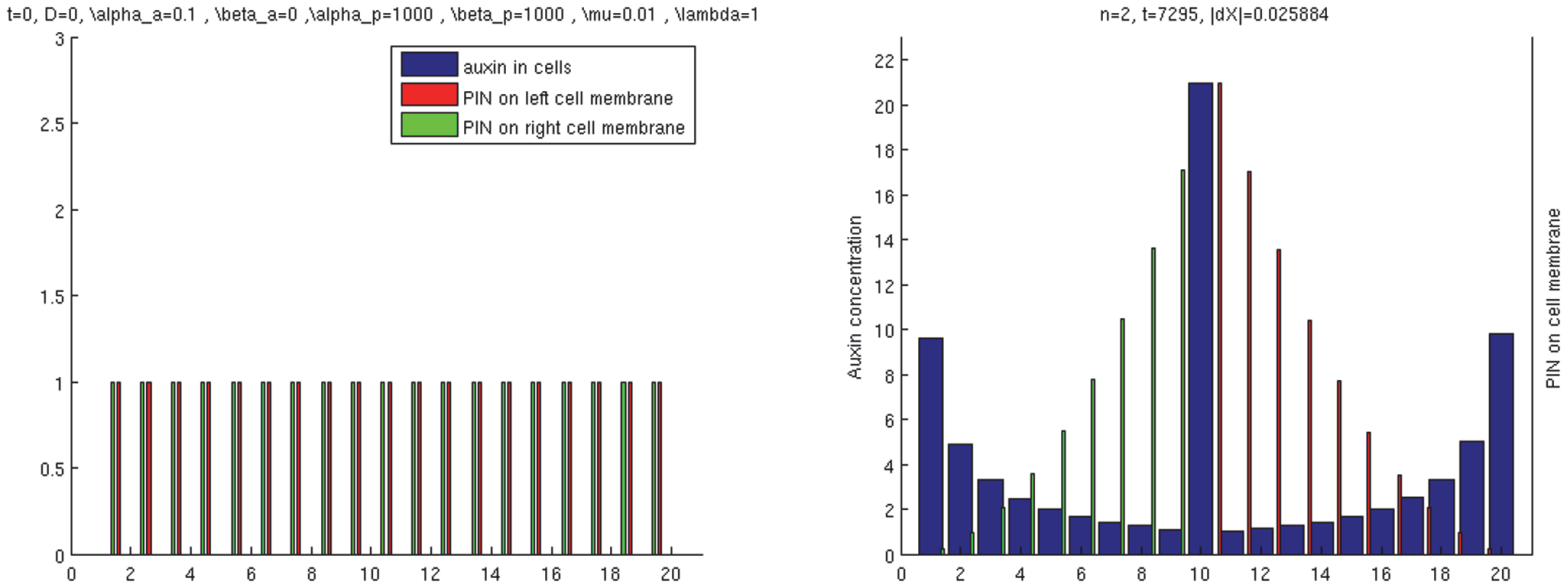
Simulation of sink-driven system with $\beta_{a}^{10}=0.1$ and $\beta_{a}^{i}=0$ for *i* ≠ *i*_0_ = 10. Notice however that the simulation does not seem to converge but instead oscillates around an equilibrium.

*i*_0_ is evacuating auxin at positive rate while the other cells (of the L1 layer) have low auxin degradation rates. Each cell *i* produces auxin at rate $\alpha_{a}^{i}\geq 0$. We will in this way obtain exact solutions when $\beta_{a}^{i}=0$, *i* ≠ *i*_0_, that permit to determine if really, within the modelling framework given by (St0), auxin is depleted in surrounding cells. When *i* ≠ *i*_0_ is such that $\beta_{a}^{i}=0$ and $a_{i}^{*}<+\infty$, then necessarily *i* ∉ I*. We will show in the S1 Text that *i*_0_ must be the unique sink cell. Hence, the oriented forest ${𝓖}^{*}$ reduces to a directed spanning tree rooted at *i*_0_. Let ${𝓖}_{i}^{*}$ be the directed rooted sub-tree of ${𝓖}^{*}$ that points to *i*, of node set $V_{i}^{*}$ (see Fig. 6), and let

$$\begin{array}{c} \alpha_{a}\left(i\right)=\sum_{j\in V_{i}^{*}}\alpha_{a}^{j}, \end{array}$$

be the global auxin production rate associated with the sub-tree ${𝓖}_{i}^{*}$. We show in the S1 Text that the **steady state auxin concentrations are given by the exact formulas**

$$\begin{array}{ccc} a_{i} & = & \frac{1}{c\alpha_{a}\left(i\right)},\;i\neq i_{0}. \end{array}$$ (4)

$$\begin{array}{ccc} a_{i_{0}} & = & \frac{\alpha_{a}\;\left(i_{0}\right)}{\beta_{a}^{i_{0}}}, \end{array}$$ (5)

where we *c* = *λα*_*p*_/(*μβ*_*p*_). Starting from a source node of the rooted tree ${𝓖}^{*}$, the sums *α*_*a*_(*i*) increase along the unique path to *i*_0_: one deduces then that the auxin concentration decreases along the paths as long as *i* ≠ *i*_0_, so that the paths are directed against the auxin gradients (see Figs. 6 and 7). This also implies the existence of auxin depleted zones in the neighbourhood of the primordium *i*_0_.

### Source-driven system

As stated in the Introduction, experiments show that the primordia of the L1 layer later act as auxin sources from where the internal vascular system initiates. We study here the vascular patterns predicted by the flux-based model when there is an isolated primordium. This model has been criticized because of a perceived inability to produce high auxin concentrations inside initiating veins, see, e.g, [23] and the references therein [9, 28]. Contrary to this claim, we will prove that the model can correctly predict auxin concentrations. In the following, we assume that there is a primordium located at *i*_0_, with auxin production rate higher than that of the other cells. Mathematically, we assume here that

$$\begin{array}{c} \beta_{a}^{i}=\beta >0,\;\forall i\in V,\;\alpha_{a}^{i}=\alpha >0,\;\forall i\neq i_{0}, \end{array}$$

and that

$$\begin{array}{c} \alpha_{a}^{i_{0}}=\alpha_{0}\geq 2\sqrt{\frac{\beta }{c}}>\alpha . \end{array}$$ (6)

As ${𝓖}^{*}$ is composed of oriented trees pointing to roots, it contains at least one source, which must be *i*_0_. Furthermore, we prove in the S1 Text that the fact that ${𝓖}^{*}$ is a forest composed of directed trees, with a distinguished cell *i*_0_, imply that the vein ${𝓖}^{*}$ must be a linear chain

$$\begin{array}{c} i_{0}⟶i_{1}⟶i_{2}⟶\cdots ⟶i_{n}, \end{array}$$

that is, must be a directed line with no vascular strands ending in a sink *i*_*n*_.

**Fig 8:**
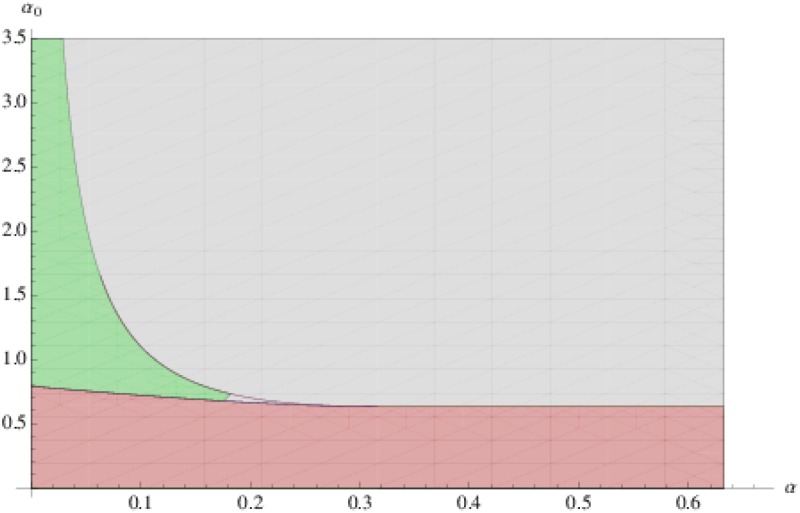
Parameter regime for a source-driven system with *c* = 1, *λ* = *μ* = 0.01 and *β* = 0.1. A: the region in green corresponds to pairs (*α*, *α*_0_) leading to flux that increase along the vein, with values that are greater than the background auxin level *α*/*β*. B: the region in gray corresponds to the reverse case where the flux decreases along the vein, taking values that are lower than the background level *α*/*β*. Surprisingly, auxin concentrations increase along the forming vein when the source production rate *α*_0_ is low, whereas the auxin concentration decreases along the vein for highly productive sources *i*_0_ with high values of *α*_0_.

**Fig 9:**
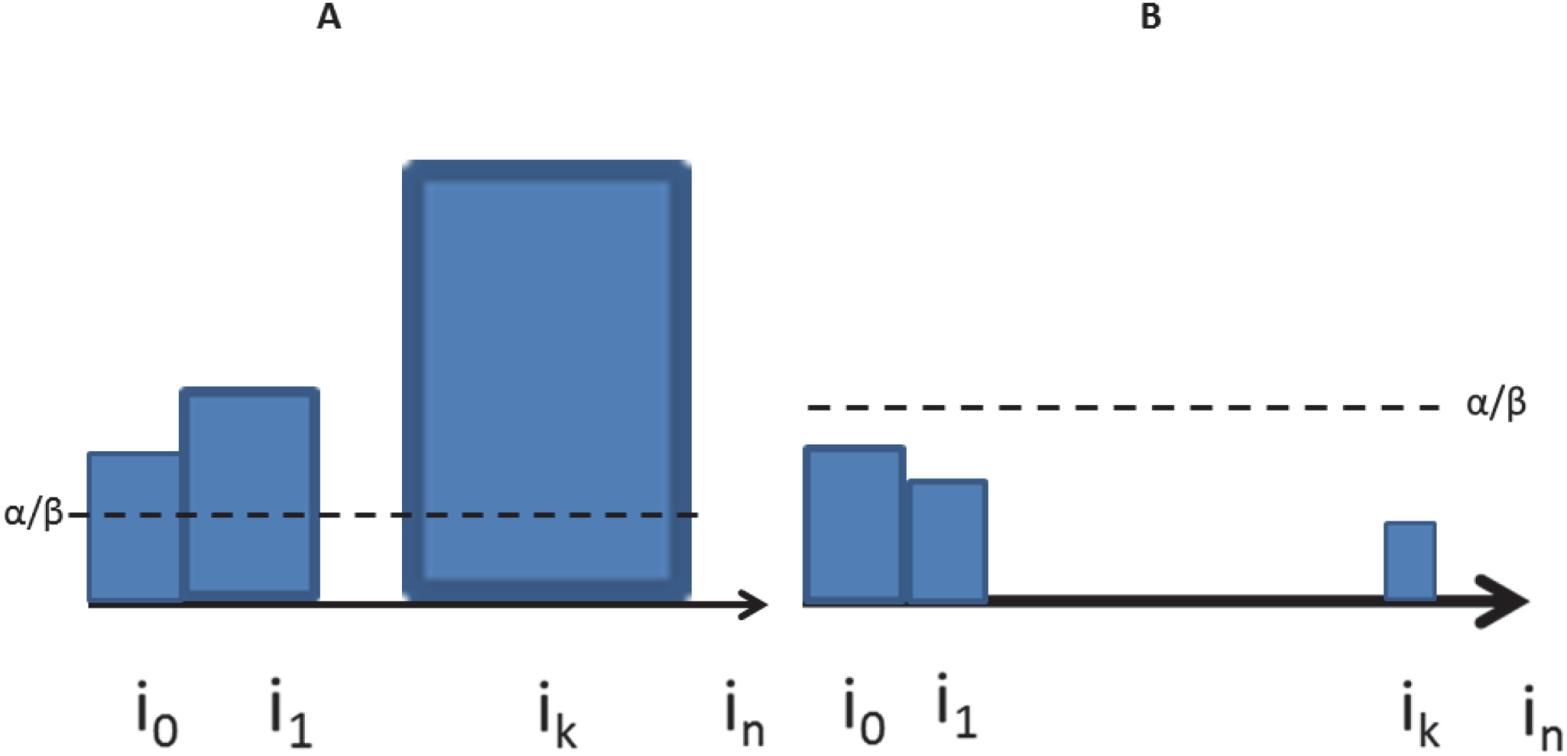
Source driven vein formation. Schematic representation of the directed auxin flux along a linear vein with the auxin gradient (A) and against the auxin gradient (B). *α*/*β* gives the background auxin concentration. The sizes of the blue boxes are proportional to auxin concentration.

We can distinguish two main parameter regimes, leading to different kinds of flux. In the first case, the auxin concentrations satisfy

$$\begin{array}{c} \left(A\right):\;\frac{\alpha }{\beta }\leq a_{i_{0}}\leq a_{i_{1}}\leq \cdots \leq a_{i_{n}}, \end{array}$$

(see Figs. 6, 8 and 9), so that the auxin flux inside the linear vein goes along the auxin gradient. In the second case it flows against the gradient

$$\begin{array}{c} \left(B\right):\frac{\alpha }{\beta }\geq a_{i_{0}}\geq a_{i_{1}}\geq \cdots \geq a_{i_{n}}, \end{array}$$

(see Figs. 8 and 9). We provide precise mathematical formulas defining the parameter regimes leading to (A) and (B) in the S1 Text.

**Fig 10:**
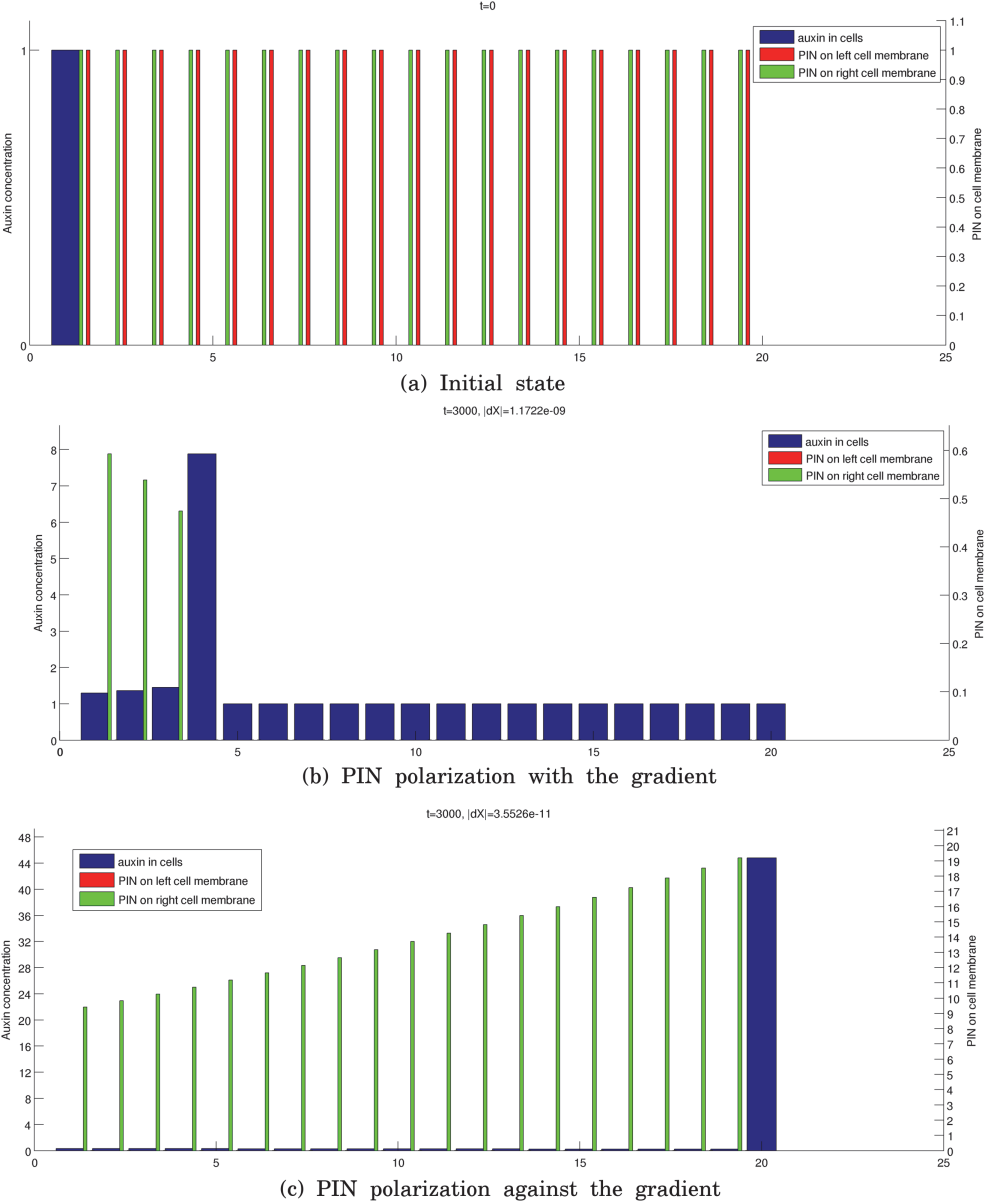
Simulation of a source-driven system. a) gives the initial state. b) and c) give the final states when $\alpha_{a}^{1}=0.9$ and $\alpha_{a}^{1}=3.1$. In c), the auxin concentration decreases gradually from cell 1 to cell 19, and is always lower than 1.

Simulations of these two kinds of source-driven system are provided in Fig. 10. We consider a line of *L* = 20 cells (without periodic boundary conditions) with *i*_0_ = 1 and take

$$\begin{array}{c} \alpha =\beta =0.1,\;\beta_{a}^{1}=0.1,\;\lambda =\mu =0.01, \end{array}$$

so that $\beta >\frac{\mu }{2}$. To recover the two regimes (see Fig. 8), we take a) $\alpha_{a}^{1}=0.9$ leading to PIN polarization with the auxin gradient and a higher auxin level in the canal than in the surroundings and b) $\alpha_{a}^{1}=3.1$ resulting in PIN polarization against the auxin gradient and a lower auxin level in the canal than in the surrounding isolated cells.

Thus, contrary to what has been claimed in previous studies, see, e.g., [23] and the references therein, the flux-based model does not necessarily lead to auxin concentrations within canals that are lower than the background concentration *α*/*β* associated with isolated cells. Instead, the resulting configuration depends on the source-strength of *i*_0_ ($\alpha_{a}^{i_{0}}$) relatively to the other cells (i.e *α*). One also observes the following counter-intuitive and surprising result: the flux increases along the vein when the production rate *α*_0_ of the source is low, whereas the flux decreases for highly productive sources with large *α*_0_.

## Discussion

We have studied various aspects of the flux-based auxin transport computational model. The model has been criticized because numerical simulations indicate that auxin concentrations within initiating veins are lower than those seen experimentally. Various extensions have been proposed to overcome this problem. We have proved that contrary to what is claimed in the literature, the flux-based model is able to correctly reproduce auxin concentrations within veins. A mathematical analysis permits us to give in a precise way the related parameter regimes. Our analyses show that for quadratic and unbounded response functions Φ, the steady state vascular patterns are formed of directed trees and thus do not contain loops. Moreover, we have proven that PIN concentrations can diverge in finite time, explaining in this way why previous simulation studies were forced to introduce cut-offs to ensure that PIN concentrations remained bounded. On the other hand, we showed that PIN concentrations remain bounded for bounded Φ; simulations suggest that in this case, loops can emerge at steady state. Thus, according to this model, the self-organization of the auxin flux leads to different patterns as a function of the boundedness of Φ. Plants having a tree-like vascular system should therefore be modelled using unbounded Φ, at the risk of recovering unrealistically high values of PIN concentration. Finally, our analysis of source- and sink-driven systems reveals new and surprising results: for sink- driven systems, the auxin flux is directed against auxin gradients, leading to an auxin depleted zone in the neighbourhood of the primordium, while for source-driven systems, a linear vein emerges which can have high auxin concentration and be directed with the auxin gradient. Surprisingly, the latter situation occurs when the auxin production rate of the source is relatively low. This model is thus able to reproduce phylotactic pattern and canalization by varying auxin synthesis and degradation rate in each cell. The remaining question is why a cell becomes a sink or source.

## Supporting Information

S1 Text (PDF)
