# Supplementary material for: Self-Organization of Plant Vascular Systems: Claims and Counter-Claims about the Flux-Based Auxin Transport Model
Source: PLoS One. 2015 Mar 3;10(3):e0118238. doi: 10.1371/journal.pone.0118238 (PMC4348546; doi:10.1371/journal.pone.0118238)
Supplement: S1 Text — (PDF) [file pone.0118238.s001.pdf]

Self-organization of plant vascular systems: claims and counter-claims about the flux-based auxin transport model

Supplementary Text

C. Feller, E. Farcot, C. Mazza

Consider the system (3) of the main text

$$\begin{aligned}\frac{da_i}{dt} &= \alpha_a^i - \beta_a^i a_i - \sum_{j\sim i}\left(D(a_i - a_j) + T(a_i p_{ij} - a_j p_{ji})\right) \\ \frac{dp_{ij}}{dt} &= \lambda P_i \Phi(J_{i\to j}) - \mu p_{ij} \\ \frac{dP_i}{dt} &= \alpha_p - \beta_p P_i + W\sum_{j\sim i}(\mu p_{ij} - \lambda P_i \Phi(J_{i\to j}))\end{aligned} \tag{1}$$

where $J_{i\to j} = D(a_j - a_i) + T(a_i p_{ij} - a_j p_{ji})$ is the net flux between cell i and cell j.

Assume that

$$\alpha_p = \frac{\alpha_p^0}{\varepsilon},\ \beta_p = \frac{\beta_p^0}{\varepsilon} \text{ and } D = D^0\varepsilon,$$

for a small parameter $\varepsilon > 0$. The main text claims that the system (1) is asymptotically equivalent as $\varepsilon \to 0$ to the simplified system

$$\begin{aligned}\frac{da_i}{dt} &= \alpha_a^i - \beta_a^i a_i + \sum_{k\sim i} T(a_k p_{ki} - a_i p_{ik}), \quad \text{for } i \in V \\ \frac{dp_{ij}}{dt} &= \lambda \frac{\alpha_p}{\beta_p}\Phi(T(a_i p_{ij} - a_j p_{ji})) - \mu p_{ij}, \quad \text{for } i,j \in V,\ i \sim j\end{aligned} \tag{St0}$$

We will provide more information on this limiting process in what follows, assuming for notational convenience that $T = 1$.

S.1 Model simplification

Theorem S.1.1 below shows that for any solution $\bar{\boldsymbol{x}}(t) = (\bar{\boldsymbol{a}}(t), \bar{\boldsymbol{p}}(t))$ of (St0) converging towards an equilibrium, the related perturbed solution resulting from the addition of small enough diffusion will remain asymptotically in a small neighbourhood of $\bar{\boldsymbol{x}}$.

Theorem S.1.1 *Let $(\bar{a}(t), \bar{p}(t))$ be the unique solution of the reduced problem (St0) for the initial conditions $(\boldsymbol{a}_0, \boldsymbol{p}_0) \in (0,\infty)^M \times (0,\infty)^m$. Let $\bar{P}(t)$ be the unique solution of the boundary layer equation*

$$\frac{d\boldsymbol{P}}{dt}(t) = \alpha_p^0 - \beta_p^0 P_i,\ \boldsymbol{P}(0) = \boldsymbol{P}_0$$

where $\boldsymbol{P}_0 > 0$. Assume moreover that $(\boldsymbol{a}_0, \boldsymbol{p}_0)$ lies in the basin of attraction of a locally asymptotically stable equilibrium point of the slow equation (St0). Then, for every $\nu > 0$, there exists $\delta > 0$ such that,

for any $\varepsilon < \delta$, any solution $(a^\epsilon(t), p^\epsilon(t), P^\epsilon(t))$ of the perturbed problem (1), which starts with the same initial condition, is defined for every $t \geq 0$, and there exists $t^* > 0$ (depending of ϵ) such that $\epsilon t^* < \nu$ and

$$||a^\epsilon(t) - \bar{a}(t)|| < \nu, \text{ for } t \geq 0,$$

$$||p^\epsilon(t) - \bar{p}(t)|| < \nu, \text{ for } t \geq 0,$$

$$||P^\epsilon(\epsilon t) - \bar{P}(t)|| < \nu, \quad \text{for } 0 \leq t \leq t^*,$$

$$||P^\epsilon(t) - \alpha_p/\beta_p|| < \nu, \text{ for } t \geq \epsilon t^*,$$

The proof is a consequence Tykhonov's Theorem (1952), see, e.g. Lobry et al. (1998).

S.2 Uniqueness and interval of definition of solution of (St0)

We study the reduced system (St0) which corresponds to Stoma's model (2008) without diffusion:

$$\begin{aligned} \frac{da_i}{dt} &= \alpha_a^i - \beta_a^i a_i + T \sum_{k \sim i} (a_k p_{ki} - a_i p_{ik}) = G_i(\boldsymbol{a}, \boldsymbol{p}) \\ \frac{dp_{ij}}{dt} &= \lambda \frac{\alpha_p}{\beta_p} \Phi(J_{i \to j}) - \mu p_{ij} = F_{ij}(\boldsymbol{a}, \boldsymbol{p}) \end{aligned} \tag{2}$$

for $i, j \in V$, $i \sim j$.

Since F and G are continuous in $C^0(\mathbb{R}^{M+m}, \mathbb{R}^M)$ resp. $C^0(\mathbb{R}^{M+m}, \mathbb{R}^m)$, the general theory of o.d.e.'s provides the existence of a solution defined over a right maximal interval $0 \in J \subset \mathbb{R}_+$ for any initial condition $(\boldsymbol{a}_0, \boldsymbol{p}_0) \in \mathbb{R}^{M+m}_{\geq 0}$. Moreover, the solution is unique because F is locally Lipschitz as Φ is locally Lipschitz.

Let $(\boldsymbol{a}(t), \boldsymbol{p}(t))$ be the solution of (2) with initial condition $(\boldsymbol{a}_0, \boldsymbol{p}_0) \in R^{M+m}_{>0}$. Assume there exists a time $t^* \in J$ such that a component of the solution $(\boldsymbol{a}(t), \boldsymbol{p}(t))$ reaches 0 in t^*. Let $\delta \in J$ be the first time it happens i.e. $a_i(\delta) = 0$ for a certain i or $p_{ij}(\delta) = 0$ for $i \sim j$. Assume the first possibility is true then

$$\frac{da_i}{dt}(\delta) = \alpha_a^i - \beta_a^i a_i(\delta) + T \sum_{k \sim i} (a_k(\delta) p_{ki}(\delta) - a_i(\delta) p_{ik}(\delta)) = \alpha_a^i + \sum_{k \sim i} T a_k(\delta) p_{ki}(\delta) \geq \alpha_a^i > 0$$

which is a contradiction with $a_i(t) > 0, \forall t \in [0, \delta)$ and $a_i(\delta) = 0$. As a consequence, there exist $i, j \in V$ such that $i \sim j$ and $p_{ij}(\delta) = 0$. On the other hand, as

$$\frac{dp_{ij}}{dt} = \lambda \frac{\alpha_p}{\beta_p} \Phi(J_{i \to j}) - \mu p_{ij}$$

then

$$p_{ij}(t) = e^{-\mu t} \left(p_{ij}(0) + \int_0^t \lambda \frac{\alpha_p}{\beta_p} \underbrace{\Phi(J_{i \to j}(\tau))}_{\geq 0} e^{\mu \tau} d\tau \right) > 0, \ \forall t \in J$$

as $p_{ij}(0) > 0$. Thus $p_{ij}(\delta)$ cannot be zero.

In what follows, we suppose that $\beta_a^i > 0$, $\forall i \in V$. Assume there exists $\delta < \infty$ such that $|(\boldsymbol{a}(t), \boldsymbol{p}(t))| \to \infty$ as $t \to \delta$ and Φ is bounded i.e. there exists $N > 0$ such that $0 \le \Phi(x) < N$ for every $x \in \mathbb{R}$. As

$$\begin{aligned} 0 < p_{ij}(t) &= e^{-\mu t}\left(p_{ij}(0) + \int_0^t \lambda \frac{\alpha_p}{\beta_p} \underbrace{\Phi(J_{i\to j}(\tau))}_{0\le\cdots\le N} e^{\mu\tau} d\tau\right) \\ &\le e^{-\mu t}\left(p_{ij}(0) + N e^{\mu t}\int_0^t \lambda \frac{\alpha_p}{\beta_p} d\tau\right) \\ &\le p_{ij}(0)e^{-\mu t} + N\lambda\frac{\alpha_p}{\beta_p} < \infty, \ \forall t \in J \end{aligned}$$

i.e. every p_{ij} is bounded in J. As there exists $\delta < \infty$ such that $|(\boldsymbol{a}(t), \boldsymbol{p}(t))| \to \infty$ as $t \to \delta$, it implies that $\sup_{t\in J} a_i(t) = \infty$ for at least one $i \in V$ and thus $\sup_{t\in J} \sum_i a_i(t) = \infty$, i.e there exists a sequence $(t_n)_{n\in\mathbb{N}}$ in J with $t_n \uparrow \delta$ such that $\sum_i a_i(t_n) \uparrow \infty$ as $n \to \infty$. It implies the existence of a sequence $(s_n)_{n\in\mathbb{N}}$ in J such that $s_n \uparrow \delta$, $\sum_i a_i(s_n) \to \infty$ as $n \to \infty$ and $\sum_i \dot{a}_i(s_n) > 0$ for every $n \in \mathbb{N}$. But:

$$\begin{aligned} \sum_i \frac{da_i}{dt}(s_n) &= \sum_i \left(\alpha_a^i - \beta_a^i a_i(s_n) + T\sum_{k\sim i}(a_k(s_n)p_{ki}(s_n) - a_i(s_n)p_{ik}(s_n))\right) \\ &= \underbrace{\sum_i \alpha_a^i}_{<\infty} - \underbrace{\sum_i \beta_a^i a_i(s_n)}_{\to\infty} \to -\infty, \text{ as } n \to \infty, \end{aligned}$$

which contradicts $\sum_i \dot{a}_i(s_n) > 0$ for every $n \in \mathbb{N}$. As a consequence, $J = [0, \infty)$ if Φ is bounded and $\beta_a^i > 0$, $\forall i \in V$.

S.3 Divergence in finite time

We here provide a simple example where the exact solution can be given in closed form: Assume that the graph $\mathcal{G}$ is a circle, and that $\Phi(x) = x^2 I_{x\ge 0}(x)$. Set

$$a_i(0) = a_0, \ p_{i,i+1}(0) = p_0^+ \text{ and } p_{i,i-1}(0) = p_0^-,$$

with $p_0^+ > p_0^- \ge 0$. From symmetry, $a_i(t) = a_j(t) \equiv a(t)$, $p_{i,i+1}(t) = p_{j,j+1}(t) \equiv p^+(t)$ and $p_{i,i-1}(t) = p_{j,j-1}(t) \equiv p^-(t)$. Suppose that $\alpha_a^i = \alpha_a$, $\beta_a^i = \beta_a > 0$, $\forall i \in V$ and $a_0 = \frac{\alpha_a}{\beta_a}$. Then,

$$a(t) = \frac{\alpha_a}{\beta_a} + \left(a_0 - \frac{\alpha_a}{\beta_a}\right)e^{-\beta_a t} = a_0, \ p^-(t) = p_0^- e^{-\mu t},$$

and

$$p^+(t) = p_0^- e^{-t\mu} - \frac{(p_0^- - p_0^+)e^{-t\mu}}{1 + a_0^2 c(p_0^- - p_0^+) - a_0^2 c(p_0^- - p_0^+)e^{-t\mu}},$$

when $p_0^+, p_0^- \ge 0$ and where

$$c := \frac{\lambda}{\mu}\frac{\alpha_p}{\beta_p}. \tag{3}$$

If the difference in the initial PIN concentration between the left and right membrane is high enough, that is, if $p_0^+ - p_0^- > \frac{1}{a_0^2 c}$, the function $p^+(t)$ is well-defined when

$$t \ne \delta = \frac{1}{\mu}\ln\left(\frac{a_0^2 c(p_0^+ - p_0^-)}{a_0^2 c(p_0^+ - p_0^-) - 1}\right) > 0.$$

In that case, $p^+(t)$ is thus only defined on $[0, \delta)$ since $p^+(t) \to \infty$ when $t \to \delta$.

S.4 A Poisson equation to describe critical points of (St0)

Let $\mathcal{G}^*$ be an oriented sub-graph of G of the type defined previously. For quadratic response functions $\Phi(x) = x^2 I_{\{x\geq 0\}}(x)$, the equilibria of (St0) can be studied using a Poisson equation, which is defined using the Laplace operator associated with the oriented graph $\mathcal{G}^*$

$$\mathcal{L}_{ij} = -I_{i\to j} \text{ if } j \sim i \text{ and } \mathcal{L}_{ii} = \sum_{j\leftarrow i} 1 = -\sum_{j\neq i} \mathcal{L}_{ij}, \tag{4}$$

where $I_{i\to j} = 1$ if the directed edge $(i \to j)$ belongs to the edge set of $\mathcal{G}^*$, and vanishes otherwise. We look for an equilibrium $X = (\boldsymbol{a}, \boldsymbol{p})$ of (St0): we will show in what follows that X is an equilibrium if and only if

- $p_{ij} = \frac{1}{ca_i^2} I_{i\to j}, \forall i, j,$
- $\boldsymbol{a}$ is obtained by solving the following Poisson equation

$$\mathcal{Q}^T b = c\beta_a \frac{1}{b} - \alpha_a c, \tag{5}$$

  where $\boldsymbol{b} = \boldsymbol{1}/a$, $\mathcal{Q} = -\mathcal{L}$.

This holds true since $(\boldsymbol{a}, \boldsymbol{p})$ is a non-negative critical point of (St0) with $\boldsymbol{a} > 0$ and associated directed sub-graph $\mathcal{G}^*$ if and only if

$$\begin{aligned}
\beta_a^i a_i &= \alpha_a^i + \sum_{j\to i} \frac{1}{ca_j} - \sum_{j\leftarrow i} \frac{1}{ca_i} \\
\Leftrightarrow \quad & c\left(\beta_a^i \frac{1}{b_i} - \alpha_a^i\right) = \sum_{j\to i} b_j - \sum_{j\leftarrow i} b_i = \sum_j \mathcal{Q}_{ji} b_j + \mathcal{Q}_{ii} b_i \\
\Leftrightarrow \quad & (\mathcal{Q}^T b)(i) = c\left(\beta_a^i \frac{1}{b_i} - \alpha_a^i\right)
\end{aligned}$$

where $\boldsymbol{b} = \boldsymbol{1}/a$, $\mathcal{Q} = -\mathcal{L}$ and $\mathcal{L}$ the following Laplace operator associated to $\mathcal{G}^*$:

$$\mathcal{L}_{ij} = -I_{i\to j} \text{ if } j \sim i \text{ and } \mathcal{L}_{ii} = \sum_{j\leftarrow i} 1 = -\sum_{j\neq i} \mathcal{L}_{ij}.$$

S.5 Locally asymptotically stable configurations for quadratic response functions

We recall the Instability result provided in the Main Text, the proof of which is given in what follows:

Theorem S.5.1 *Consider the system (St0) with $\Phi(x) = x^2$. Assume that $\alpha_a^i, \mu, c > 0$, and $\beta_a^i \geq 0$, $\forall i \in V$. Let $(\boldsymbol{a}^*, \boldsymbol{p}^*) \in (0, \infty)^M \times (0, \infty)^m \backslash E_0$ be an equilibrium of the system (St0) of associated oriented sub-graph $\mathcal{G}^*$. Then,*

- *If $\mathcal{G}^*$ contains no sink cells, then $(\boldsymbol{a}^*, \boldsymbol{p}^*)$ is unstable.*

- *If $(\boldsymbol{a}^*, \boldsymbol{p}^*)$ is stable, then $\mathcal{G}^*$ is an oriented sub-graph of G composed of trees directed from leaves to roots such that every cell $i \in V$ has at out-degree ≤ 1.*

Theorem S.5.1 shows that the possibly stable equilibria of (St0) are such that $\mathcal{G}^*$ is composed of tree directed from leaves to the root such that every cell i has at most one descendent, i.e., $\sharp\{k \leftarrow i\} \leq 1$, containing therefore at least one source and one sink cell. The Poisson equation (5) rewrites as

$$\sum_{j\to i} \frac{1}{a_j} - \frac{1}{a_i} = c\beta_a^i a_i - \alpha_a^i c, \tag{6}$$

when i is not a sink, while

$$\sum_{j\to i} \frac{1}{a_j} = c\beta_a^i a_i - \alpha_a^i c, \tag{7}$$

for sink cells i.

When $\beta_a^i > 0$, $\forall i \in V$, we deduce from (7) that auxin concentration at a sink cell is obtained from the formula

$$a_i = \frac{\alpha_a^i + \sum_{j\to i} \frac{1}{ca_j}}{\beta_a^i}. \tag{8}$$

We get a finite number of equilibria, which can be computed recursively from the source cells (choosing one of the two above values for each source) to the sink cells by using the formula

$$a_i = \frac{\alpha_a^i + \sum_{j\to i} \frac{1}{ca_j} \pm \sqrt{(\alpha_a^i + \sum_{j\to i} \frac{1}{ca_j})^2 - 4\beta_a^i \frac{1}{c}}}{2\beta_a^i}$$

if

$$(\alpha_a^i + \sum_{j\to i} \frac{1}{ca_j})^2 - 4\beta_a^i \frac{1}{c} \geq 0, \tag{9}$$

since $\sharp\{j \leftarrow i\} = 1$.

S.6 Proof of the instability criterion

We compute first the Jacobian matrix of the system (St0) (or (2)). Let

$$\begin{aligned} G_i &= \alpha_a^i - \beta_a^i a_i + \sum_{k\sim i} (a_k p_{ki} - a_i p_{ik}) \\ F_{ij} &= \lambda \frac{\alpha_p}{\beta_p} \Phi\left(a_i p_{ij} - a_j p_{ji}\right) - \mu p_{ij} \end{aligned}$$

and $\mathcal{L}$ the matrix with coefficients:

$$\mathcal{L}_{ij} = I_{i\to j} \text{ and } \mathcal{L}_{ii} = -\sum_j I_{i\to j} = -\sharp\{j \leftarrow i\}.$$

Let Γ the adjacency matrix of G i.e. $\gamma_{ij} = 1$ if and only if $(i,j) \in E$ (i.e $i \sim j$) and $\gamma_{ij} = 0$ otherwise. We find $\left(\frac{\partial G_i}{\partial a_k}\right)_{k,i} = -diag(\beta_a^i) + diag(\frac{1}{c(a_i^*)^2})\mathcal{L}$ and $(\frac{\partial G_i}{\partial p_{kl}})_{kl,i}$ is a block matrix with blocks:

$$\begin{array}{c} \\ ij \\ ji \end{array} \begin{array}{c} \begin{array}{ccccccccccc} 1 & & & i & & & & j & & & M \end{array} \\ \begin{pmatrix} 0 & \cdots & 0 & -a_i^* & 0 & \cdots & 0 & a_i^* & 0 & \cdots & 0 \\ 0 & \cdots & 0 & a_j^* & 0 & \cdots & 0 & -a_j^* & 0 & \cdots & 0 \end{pmatrix} \end{array}$$

For F we obtain that $(\frac{\partial F_{ij}}{\partial p_{kl}})_{kl,ij}$ is a block-diagonal matrix with blocks:

$$\begin{pmatrix} \frac{\partial F_{ij}}{\partial p_{ij}} & \frac{\partial F_{ji}}{\partial p_{ij}} \\ \frac{\partial F_{ij}}{\partial p_{ji}} & \frac{\partial F_{ji}}{\partial p_{ji}} \end{pmatrix} = \begin{pmatrix} \mu & 0 \\ \star & -\mu \end{pmatrix} \text{ if } i \to j \text{ or } \begin{pmatrix} -\mu & 0 \\ 0 & -\mu \end{pmatrix} \text{ if } i \sim j \text{ but } i \not\to j \text{ and } j \not\to i$$

and $(\frac{\partial F_{ij}}{\partial a_k})_{k,ij}$ is a block matrix with vertical blocks:

$$\left(\frac{\partial F_{ij}}{\partial a_k} \; \frac{\partial F_{ji}}{\partial a_k}\right)_{k=1,\cdots,L} = \begin{pmatrix} 0 & 0 \\ \vdots & \vdots \\ 0 & 0 \\ \frac{2\mu}{c(a_i^*)^3} I_{i\to j} & 0 \\ 0 & 0 \\ \vdots & \vdots \\ 0 & 0 \end{pmatrix}$$

In summary, the Jacobian matrix J is: $J^T = \begin{pmatrix} \frac{\partial F_{ij}}{\partial p_{kl}} & \frac{\partial G_i}{\partial p_{kl}} \\ \frac{\partial F_{ij}}{\partial a_k} & \frac{\partial G_i}{\partial a_k} \end{pmatrix}_{i,j,k,l \in V, i \sim j, k \sim l}$

$$
J^T = \left(\begin{array}{c|cccc|cccccccccccc}
 & & ij & ji & & & & & i & & & & j & & & k & l \\
 & \ddots & & & & & & & & & & & & & & & \\
L_{ij} & & \mu & 0 & & 0 & \cdots & 0 & -a_i^* & 0 & \cdots & 0 & a_i^* & 0 & \cdots & 0 & 0 \\
L_{ji} & & -2\mu\frac{a_j^*}{a_i^*} & -\mu & & 0 & \cdots & 0 & a_j^* & 0 & \cdots & 0 & -a_j^* & 0 & \cdots & 0 & 0 \\
 & & & & \ddots & & & & & & & & & & & & \\ \hline
 & \cdots & 0 & 0 & \cdots & & & & & & & & & & & & \\
 & & \vdots & \vdots & & & & & & & & & & & & & \\
L_i & \cdots & \frac{2\mu}{c(a_i^*)^3} & 0 & \cdots & & & & \beta_a^i - \frac{\sharp\{l \leftarrow i\}}{c(a_i^*)^2} & & & & \frac{1}{c(a_i^*)^2} & & & \frac{1}{c(a_i^*)^2} & \\
 & & \vdots & \vdots & & & & & & & & & & & & & \\
 & & 0 & 0 & & & & & & & & & & & & & \\
L_j & & 0 & 0 & & & & & 0 & & & & \beta_a^j - \frac{\sharp\{l \leftarrow j\}}{c(a_j^*)^2} & & & & \frac{1}{c(a_j^*)^2} \\
 & & 0 & 0 & & & & & & & & & & & & & \\
 & & \vdots & \vdots & & & & & & & & & & & & & \\
 & & 0 & 0 & & & & & & & & & & & & &
\end{array}\right) \qquad (10)
$$

where $i \to j$, $i \to k$ and $j \to l$. Note that to simplify the presentation the following terms are not shown above but are non-zero in general:

- on row L_i the ik^{th} term is equal to $\frac{2\mu}{c(a_i^*)^3}$ as $i \to k$,
- on row L_j the jl^{th} term is equal to $\frac{2\mu}{c(a_j^*)^3}$ as $j \to l$.

Note also that for an isolated cell i, for any j with $i \sim j$ the rows L_{ij}, L_{ji}, L_i and L_j differ from above as follows:

- the 2×2 block on rows and columns (ij, ji) is $\begin{pmatrix} -\mu & 0 \\ 0 & -\mu \end{pmatrix}$ instead of $\begin{pmatrix} \mu & 0 \\ -2\mu\frac{a_j^*}{a_i^*} & -\mu \end{pmatrix}$.
- the only non-zero term of row L_i is the ith and it is β_a^i instead of $\beta_a^i - \frac{\sharp\{l \leftarrow i\}}{c(a_i^*)^2}$.

We can observe that the lower left block $\left(\frac{\partial F_{ij}}{\partial a_k}\right)_{k\in V,\, ij\in E}$ of J^T is non-zero only at entries of the form (i, ij) where i and j are such that $i \to j$. In particular, for any i without successor (i.e. i is a sink or an isolated cell), the first m terms of row L_i are zero.
For any i with a successor j_i, on the other hand, the first m entries of row ij_i are zero except the diagonal entry ij_i which is equal to μ. Hence, the rows ij_i of the matrix $J^T - \mu I$ are of the form:

$$L_{ij_i} - \mu\delta_{ij_i} = (0, \cdots, 0|0, \cdots, 0, -a_i^*, 0, \cdots, 0, a_i^*, 0, \cdots, 0).$$

So, if for all i with successors we choose one of them, say j_i, and exchange the rows $L_i - \mu\delta_i$ and $L_{ij_i} - \mu\delta_{ij_i}$ of the matrix $J^T - \mu I$ we obtain a block triangular form

$$\tilde{J} = \left(\begin{array}{c|c} A & \star \\ \hline 0 & B \end{array}\right) \qquad \text{of dimensions} \qquad \left(\begin{array}{c|c} m\times m & m\times M \\ \hline M\times m & M\times M \end{array}\right) \tag{11}$$

where the matrix B is given by

$$B_{ik} = -a_i^*\delta_{ik} + a_i^*\delta_{j_ik}, \qquad \text{if } \sharp\{k \leftarrow i\} \neq 0, \tag{12}$$

$$\begin{aligned} B_{ik} &= \left(-\beta_a - \frac{1}{c(a_i^*)^2}\sharp\{k \leftarrow i\} - \mu\right)\delta_{ik} + \sum_j \frac{1}{c(a_i^*)^2} I_{i\to j}\delta_{jk} \\ &= (-\beta_a - \mu)\delta_{ik}, \qquad \text{if } \sharp\{k \leftarrow i\} = 0. \end{aligned} \tag{13}$$

Notice that we chose a single j_i for every i with successors and that different choices will lead to different matrices A and B. However, we will show the following.

Proposition S.6.1 *Assume that every non-isolated $i \in V \setminus I^*$ has at least a successor (i.e. there is no sink). Then, for any choice of successors j_i, the resulting matrix B is singular.*

Let the vector $\boldsymbol{v} \in \mathbb{R}^M$ be defined by $v_i = 1$ for $i \in V \setminus I^*$ and $v_i = 0$ for $i \in I^*$. Since we are considering an equilibrium different from E_0, there must be at least one non-isolated cell and thus $\boldsymbol{v}$ is non-zero. Also, in the absence of sink for any $i \in V \setminus I^*$ there exists a j_i such that $i \to j_i$. For any choice of j_i indices, the corresponding matrix B satisfies

$$(B\boldsymbol{v})_i = \sum_k(-a_i^*\delta_{ik} + a_i^*\delta_{j_ik}) = -a_i^* + a_i^* = 0, \qquad \forall i \in V \setminus I^*$$

and for $i \in I^*$ the ith row of B is zero and thus $(B\boldsymbol{v})_i = 0$. In other terms, we have $B\boldsymbol{v} = \mathbf{0}$ and thus B is singular. □

It follows that $|\det(J^T - \mu I)| = |\det(A)||\det(B)| = 0$, and thus μ is an eigenvalue of J and $(\boldsymbol{a}^*, \boldsymbol{p}^*)$ is unstable.

We will now prove the following result:

Proposition S.6.2 *For a set of parameters of Lebesgue measure 1, μ is an eigenvalue of the Jacobian matrix J at $(\boldsymbol{a}^*, \boldsymbol{p}^*)$ if and only if at least one of the two following assertions is true:*

(i) $\mathcal{G}^*$ contains subgraph γ which is an oriented cycle.

(ii) There exists a cell i with at least two successors, i.e. $\sharp\{k \in V \mid k \leftarrow i\} \geq 2$.

In fact we will show that there exists a choice of successors j_i to non-sink nodes i such that the block-triangular matrix (11) verifies $\det(B) = 0 \iff$ the condition (i) is satisfied, while there exists a choice such that $\det(A) = 0 \iff$ (ii) is satisfied.

To begin with, we prove the direction "$\Leftarrow$" of the first equivalence.

Let $I_\gamma = \{i_1, \cdots i_n\}$ be the node set of the cycle γ. For each $i_k \in I_\gamma$, we chose the successor $j_{i_k} = i_{k+1}$ where i_{n+1} denotes i_1. For every other node i with a successor, we chose $j_i \leftarrow i$ arbitrarily. Let us now define the following partition of V into three node sets:

$$I_0 = I_\gamma, \qquad I_1 = I^*, \quad \text{and} \quad I_2 = V \setminus (I^* \cup I_\gamma),$$

where in general I_1 and I_2 may be empty sets.

From the construction of B, a non-diagonal element B_{ij} is non-zero if and only if $j = j_i$, and there is exactly one j_i for each $i \in I_0 \cup I_2$ and none for $i \in I_1$. Furthermore, since I_0 corresponds to the cycle, we have that $j_i \in I_0$ for all $i \in I_0$. On the other hand for $i \in I_2$, j_i may be in I_0 or I_2.

It follows that if we order the nodes in such a way that all nodes I_0 come first, followed by nodes in I_1 and finally nodes in I_2, the resulting matrix B is block-triangular, of the form:

$$\left(\begin{array}{c|c|c} B_0 & 0 & 0 \\ \hline 0 & B_1 & 0 \\ \hline B_{20} & 0 & B_2 \end{array}\right)$$

Similarly to the previous proof, one can see that B_0 is singular: each row L_i contains exactly one term a_i^* off diagonal and $-a_i^*$ on the diagonal, so that the vector with all entries equal to 1 is in its kernel.
It thus follows that $\det(B) = \det(B_0)\det(B_1)\det(B_2) = 0$.

For the other direction "$\Rightarrow$", we will proceed by contraposition. Suppose that the graph $\mathcal{G}^*$ contains no cycle and let $i \mapsto j_i$ be a map selecting an arbitrary successor for any node i having at least one. The graph of this map has the same node set V as $\mathcal{G}^*$, and at most one edge (i, j_i) for each node i. It is thus a subgraph of $\mathcal{G}^*$ with out-degree ≤ 1 (see e.g. **?** for terminology on digraphs), which can be seen to be a collection of trees directed from leaves to roots, with no more than one successor per node.
These trees can be decomposed into isolated nodes and chains of the form $i_1 \mapsto i_2 \mapsto \cdots \mapsto i_n$ where $i_{k+1} = j_{i_k}$. Then, grouping each nodes from the same chain, ordered as in the chain, with an extra set for isolated nodes, we get a partition of V. This partition is not unique in general, but any choice allows to write B in a block-triangular form, where the block associated to I^* is diagonal with diagonal terms $\beta_a^i - \mu$, off-diagonal blocks correspond to chains which are incident (i.e. such that two nodes i and k from different chains have the same successor, $j_i = j_k$), and each block associated to a chain of the form $i_1 \mapsto i_2 \mapsto \cdots \mapsto i_n$ is on the diagonal and has the form:

$$\begin{pmatrix} -a_{i_1}^* & a_{i_1}^* & & 0 \\ & \ddots & \ddots & \\ & & -a_{i_{n-1}}^* & a_{i_{n-1}}^* \\ 0 & & 0 & -a_{i_n}^* \end{pmatrix}.$$

From the form above, it thus appear that B is then not only block-triangular but triangular and its determinant is a product of terms $\beta_a^i - \mu$ and $-a_i^*$.
It is easily seen from (St0) that if $a_i = 0$ then $\dot{a}_i > 0$ and thus a steady state must satisfy $a_i^* > 0$, $\forall i$.

Except for the set of parameters (of measure 0) satisfying equalities of the form $\beta_a^i = \mu$, we have $\det(B) \neq 0$.

We now prove that (ii) is equivalent to the existence of a choice of successors j_i such that $\det(A) = 0$.

As seen earlier, the matrix A can be arranged in pairs of rows, of the form

$$\begin{array}{c} \\ ij \\ ji \end{array} \begin{array}{c} \begin{array}{ccccccccc} & & & ij & ji & & & & \end{array} \\ \begin{pmatrix} 0 & \cdots & 0 & -2\mu & 0 & 0 & \cdots & 0 \\ 0 & \cdots & 0 & 0 & -2\mu & 0 & \cdots & 0 \end{pmatrix} \end{array} \quad \text{if } i \sim j \text{ but } i \not\to j \text{ and } j \not\to i,$$

$$\begin{array}{c} \\ ij_i \\ j_i i \end{array} \begin{array}{c} \begin{array}{ccccccccc} & & & ij_i & j_i i & & & & \end{array} \\ \begin{pmatrix} 0 & \cdots & 0 & \frac{2\mu}{c(a_i^*)^3} & 0 & 0 & \cdots & 0 \\ 0 & \cdots & 0 & 0 & -2\mu & 0 & \cdots & 0 \end{pmatrix} \end{array} \quad \text{for the chosen successor } j_i \leftarrow i,$$

and

$$\begin{array}{c} \\ ij \\ ji \end{array} \begin{array}{c} \begin{array}{ccccccccc} & & & ij & ji & & & & \end{array} \\ \begin{pmatrix} 0 & \cdots & 0 & 0 & 0 & 0 & \cdots & 0 \\ 0 & \cdots & 0 & -2\mu\frac{a_{j_i}^*}{a_i^*} & -2\mu & 0 & \cdots & 0 \end{pmatrix} \end{array} \quad \text{for any other } j \leftarrow i,\ j \neq j_i.$$

The indices of matrix A are all indices ij and ji for pairs $i \sim j$ of adjacent cells. For each $i \sim j$, there is exactly one non-zero entry for both ij and ji, on the diagonal, except for entries ij where $i \to j$ and $j \neq j_i$. So, clearly $\det(A) = 0$ iff the latter occurs, i.e. iff there exists i such that $\sharp\{k \in V \mid k \leftarrow i\} \geq 2$.

□

Remark S.6.3 *The Theorem remains true for $\Phi^{(\theta)}(x) = x^\theta I_{x\geq 0}$ for $\theta > 1$. In that case,*

$$F_{ij} = \lambda \frac{\alpha_p}{\beta_p} \Phi^{(\theta)}(J_{i\to j}) - \mu p_{ij} \text{ with } J_{i\to j} = a_i p_{ij} - a_j p_{ji}$$

and

$$\frac{\partial F_{ij}}{\partial a_i} = \mu \frac{\theta}{a_i^*(c(a_i^*)^\theta)^{1/(\theta-1)}} I_{i\to j} \qquad \frac{\partial F_{ij}}{\partial p_{ij}} = \theta\mu I_{i\to j} - \mu$$

$$\frac{\partial F_{ij}}{\partial a_j} = 0 \qquad \frac{\partial F_{ij}}{\partial p_{ji}} = -\theta\mu I_{i\to j} \frac{a_j^*}{a_i^*}$$

We can show that $(\theta - 1)\mu > 0$ is an eigenvalue of the Jacobian matrix at $(\boldsymbol{a}^*, \boldsymbol{p}^*)$ if and only $\mathcal{G}^*$ is not a graph composed of trees directed from leaves to the root such that every cell i has at most one output i.e. $\sharp\{k \leftarrow i\} \leq 1$. The proof is similar as for the case $\theta = 2$.

In fact, the proof of the theorem relies on the occurrence of terms equal to μ (or more generally $(\theta - 1)\mu$ for $\Phi^{(\theta)}(x) = x^\theta I_{x\geq 0}$) on the diagonal of the Jacobian matrix at steady state.

To assess whether the result would still hold for other choices of the function Φ, one can leave this function unspecified and look for cases where the same diagonal terms as above are constant. This leads to a differential equation of the form

$$c a_i^* \Phi'(a_i^* p_{ij}^*) = K \qquad \text{for a constant } K,$$

and we also have $p^*_{ij} = c\Phi(a^*_i p^*_{ij})$ from the steady state equation. Now assuming Φ locally invertible (at the steady state), the two equalities lead to

$$\frac{1}{\Phi'(a^*_i p^*_{ij})} = \left(\Phi^{-1}\right)'(\Phi(a^*_i p^*_{ij})) = \left(\Phi^{-1}\right)'(p^*_{ij}/c) = \frac{ca^*_i}{K} = \frac{1}{K}\frac{\Phi^{-1}(p^*_{ij}/c)}{p^*_{ij}/c}.$$

So, if we define the function $f : x \mapsto \Phi^{-1}\left(\frac{x}{c}\right)$ this implies $f'(p^*_{ij}) = \frac{f(p^*_{ij})}{Kp^*_{ij}}$.
If this holds for an open set of values for p^*_{ij}, the function f must satisfy the differential equation $\frac{f'(x)}{f(x)} = \frac{1}{Kx} \implies \ln f = \int \frac{1}{Kx} = \ln\left(x^{\frac{1}{K}}\right)$ +constant. In other words, f must be a function of the form $x^{\frac{1}{K}}$ and so does Φ.

S.7 Stability criterion

We know that every stable critical point $(\boldsymbol{a}^*, \boldsymbol{p}^*)$ of the system (St0) is associated to an oriented graph $\mathcal{G}^*$ composed of trees directed from leaves to the root such that every cell $i \in V$ has at most one output i.e. $\sharp\{k \leftarrow i\} \leq 1$. We will now characterize the set of such oriented graphs which give rise to a stable critical point.

Theorem S.7.1 *Sufficient stability criterion*
Consider the system (St0) with $\Phi(x) = x^2 I_{x\geq 0}(x)$, $T = 1$ and parameters $\alpha^i_a, \mu, c > 0$ but $\beta^i_a \geq 0$, $\forall i \in V$. Let $\mathcal{G}^*$ be an oriented graph composed of trees directed from leaves to the root such that every cell $i \in V$ has at most one output i.e. $\sharp\{k \leftarrow i\} \leq 1$. Assume there exists a non-negative critical point $(\boldsymbol{a}^*, \boldsymbol{p}^*)$ of the system (St0) associated the orientation $\mathcal{G}^*$, different from E_0. Then, $(\boldsymbol{a}^*, \boldsymbol{p}^*)$ is locally asymptotically stable if every non-sink/non-isolated cell $i \in V$ verifies one of two following conditions:

- $0 \leq \beta^i_a < \frac{\mu}{2}$ *and* $a^*_i < a_c := -\frac{1}{c\left(\beta^i_a - 3\mu + 2\sqrt{2}\sqrt{\mu(-\beta^i_a + \mu)}\right)}$,
- $\beta^i_a \geq \frac{\mu}{2}$ *and* $a^*_i < \sqrt{\frac{1}{c\beta^i_a}}$.

For the proof, we need the following definition.

Definition S.7.2 *Let $\mathcal{G}^*$ be an oriented graph with node set V. The ancestors of $i \in V$ are all the nodes j such that there exists a path $j \to j_1 \to j_2 \to \ldots \to j_n \to i$ from j to i. Reciprocally, i is called a successor or descendent of j.*

Proof :
Let λ be an eigenvalue of the Jacobian matrix J at $(\boldsymbol{a}^*, \boldsymbol{p}^*)$, i.e. $\det(\tilde{J}) = 0$ where we define $\tilde{J} = J^T - \lambda I$ (see the structure of J^T in (10)). Due to the assumptions about $\mathcal{G}^*$ and the proof of the Instability criterion, we know that $\lambda \neq \mu$.
For a non-sink and non-isolated cell $i \in V$ there exists a unique $j_i \in V$ with $i \to j_i$. Then, the ith and (ij)th rows of $\tilde{J}$ are almost everywhere equal to 0 except

$$(L_i)_{ij_i} = \frac{2\mu}{c(a^*_i)^3}, \qquad (L_i)_i = -\beta^i_a - \frac{1}{c(a^*_i)^2} - \lambda, \qquad (L_i)_{j_i} = \frac{1}{c(a^*_i)^2},$$
$$(L_{ij_i})_{ij_i} = \mu - \lambda, \qquad (L_{ij_i})_i = -a^*_i, \qquad (L_{ij_i})_{j_i} = a^*_i.$$

It follows that the row $\tilde{L}_i := L_i - \frac{2\mu}{c(a_i^*)^3(\mu-\lambda)} L_{ij_i}$, is zero everywhere except

$$\begin{aligned}(\tilde{L}_i)_i &= -\beta_a^i - \frac{1}{c(a_i^*)^2} - \lambda - \frac{2\mu}{c(a_i^*)^3(\mu-\lambda)}(-a_i^*) = -\beta_a^i - \lambda + \frac{\lambda+\mu}{c(\mu-\lambda)(a_i^*)^2} =: B_{ii} \\ (\tilde{L}_i)_{j_i} &= \frac{1}{c(a_i^*)^2} - \frac{2\mu}{c(a_i^*)^3(\mu-\lambda)} a_i^* = \frac{-(\lambda+\mu)}{c(\mu-\lambda)(a_i^*)^2} =: B_{ij_i}\end{aligned}$$

i.e. $B_{ii} = -\beta_a^i - \lambda - B_{ij_i}$.

If i is a sink or an isolated cell then L_i is almost everywhere equal to 0 except:

$$(L_i)_i = -\beta_a^i - \lambda =: B_{ii}$$

and we don't transform it (i.e. $\tilde{L}_i = L_i$).

After the changes of rows $\tilde{L}_i \leftarrow L_i$ above, the matrix $\tilde{J}$ becomes block-triangular, of the form:

$$\tilde{J} = \left(\begin{array}{c|c} A & \star \\ \hline 0 & B \end{array}\right), \tag{14}$$

where all the non-zero terms in B have been noted above, and A is block-diagonal with 2×2 blocks of the form:

$$\begin{array}{c} \\ ij \\ ji \end{array} \begin{array}{c} \quad ij \qquad\qquad ji \\ \begin{pmatrix} -\mu-\lambda & 0 \\ 0 & -\mu-\lambda \end{pmatrix} \end{array} \quad \text{if } i \sim j \text{ but } i \not\leftarrow j \text{ and } j \not\leftarrow i,$$

$$\begin{array}{c} \\ ij_i \\ j_i i \end{array} \begin{array}{c} \quad ij_i \qquad\qquad j_i i \\ \begin{pmatrix} \mu-\lambda & 0 \\ -2\mu\frac{a_{j_i}^*}{a_i^*} & -\mu-\lambda \end{pmatrix} \end{array} \quad \text{for the unique successor } j_i \leftarrow i,$$

Note that the block-triangular form (14) is possible only because $\lambda \neq \mu$, and thus $\det(A) = 0$ only for $\lambda = -\mu$, which is thus an eigenvalue of the Jacobian J. From the form (14), other eigenvalues are exactly characterized by the condition $\det(B) = 0$.
In the case where $B_{ij_i} > 0$ for every $i \in V$ and $j_i \leftarrow i$ (i.e. $\lambda < -\mu < 0$ or $\lambda > \mu$), then B is diagonally dominant and has a vanishing determinant only if there exists $i_0 \in V$ such that $\lambda = -\beta_a^{i_0} \leq 0$. Consequently, $-\beta_a^i$ (for $i \in V$) is an eigenvalue of the Jacobian matrix if $\beta_a^i > \mu$. Moreover, none of the eigenvalues of the Jacobian is larger than μ.

Assume now that $\lambda \in [-\mu, \mu)$ (i.e. $B_{ij_i} \leq 0$ and B is not diagonally dominant). Now, $\det(B) = 0$ iff there exists a non-zero vector $v \in \mathbb{R}^M$ such that $Bv = 0$.
For each node i without successor (i.e. i is a sink or an isolated cell) we have

$$(Bv)_i = -(\beta_a^i + \lambda)v_i = 0 \implies v_i = 0 \quad \text{or} \quad \lambda + \beta_a^i = 0,$$

so that $\lambda = -\beta_a^i$ is an eigenvalue if i is a sink or an isolated node. Then, for $\lambda \neq -\beta_a^i$ to be an eigenvalue, the non-zero coordinates of v must correspond to nodes i with a successor j_i. For such nodes,

$$(Bv)_i = B_{ii}v_i + B_{ij_i}v_{j_i} = -\left(\beta_a^i + \lambda - \frac{\lambda+\mu}{c(\mu-\lambda)(a_i^*)^2}\right)v_i - \frac{\lambda+\mu}{c(\mu-\lambda)(a_i^*)^2}v_{j_i} = 0.$$

It follows that unless

$$\beta_a^i + \lambda - \frac{1}{c(a_i^*)^2}\frac{\lambda+\mu}{\mu-\lambda} = 0, \quad \text{which implies} \qquad v_{j_i} = 0, \tag{15}$$

we have

$$v_i = \frac{-(\lambda+\mu)}{c(\beta_i^a+\lambda)(\mu-\lambda)(a_i^*)^2 - (\lambda+\mu)} v_{j_i}.$$

Hence, all ancestors of a node j_i such that $v_{j_i} = 0$ will be zero themselves unless condition (15) is satisfied.

Then $Bv = 0$ with $v \neq 0$ if and only there exists a cell $i_0 \in V$ with

$$\beta_a^{i_0} + \lambda - \frac{1}{c(a_{i_0}^*)^2}\frac{\lambda+\mu}{\mu-\lambda} = 0 \tag{16}$$

and such that for every ancestor i of i_0:

$$\beta_a^i + \lambda - \frac{1}{c(a_i^*)^2}\frac{\lambda+\mu}{\mu-\lambda} \neq 0$$

Finally, the condition (16) is verified with $\lambda \in (0,\mu)$ if and only if one of two following conditions is fulfilled:

- $0 \leq \beta_a^{i_0} < \frac{\mu}{2}$ and $a_c \geq a_{i_0}^*$,
- $\beta_a^{i_0} \geq \frac{\mu}{2}$ and $a_{i_0}^* > \sqrt{\frac{1}{c\beta_a^{i_0}}}$.

with $\lambda = 0$ if and only if $\beta_a^i > 0$ and $a_{i_0}^* = \sqrt{\frac{1}{c\beta_a^{i_0}}}$. □

S.8 Sink-driven systems

We study the stable patterns in the extreme case where there is a single primordium, which is located at some site i_0 such that

$$\beta_a^i = 0, \ \forall i \neq i_0 \text{ and } \beta_a^{i_0} > 0.$$

i_0 is evacuating auxin at positive rate while the other cells (of the L1 layer) do not degrade auxin. Each cell i produces auxin at rate $\alpha_a^i \geq 0$. We will in this way obtain exact solutions that permit to determine if really, within the modelling framework given by (St0), auxin is depleted in surrounding cells. Notice that $\beta_a^i = 0$ and $a_i^* < +\infty$ when $i \neq i_0$ implies that $i \notin I^*$, that is, the graph is a spanning forest When $i \neq i_0$, the equation (7) shows that i can't be a sink, and therefore i_0 must necessarily be the unique sink of $\mathcal{G}^*$. Let $\mathcal{G}_i^*$ be the directed rooted sub-tree of $\mathcal{G}^*$ which points to i, of node set V_i^*, and let

$$\alpha_a(i) = \sum_{j \in V_i^*} \alpha_a^j,$$

be the global auxin production rate associated with the sub-tree $\mathcal{G}_i^*$. Let $\mu_i = \frac{1}{ca_i}$. Then, for $i \neq i_0$, we deduce from (6):

$$\mu_i = \sum_{j \to i} \mu_j + \alpha_a^i,$$

and a direct computation shows that $\mu_i = \alpha_a(i)$, which shows that the **steady state auxin concentration are given by the exact formula**

$$a_i = \frac{1}{c\alpha_a(i)},\ i \neq i_0. \tag{17}$$

Starting from a source node of the rooted tree $\mathcal{G}^*$, the sums $\alpha_a(i)$ increase along the unique path to i_0: one deduces then that the auxin concentration decreases along the veins as long as $i \neq i_0$, so that the veins are directed against auxin gradients. This also implies that the existence of auxin depleted zones inside canals in the neighbourhood of the primordium i_0. Concerning i_0, the Poisson equation (8) leads to

$$a_{i_0} = \frac{\alpha_a^{i_0} + \sum_{j\to i_0} \frac{1}{ca_j}}{\beta_a^{i_0}} = \frac{\alpha_a(i_0)}{\beta_a^{i_0}}.$$

S.9 Source-driven system

We assume that there is a distinguished cell i_0, like a primordium, whose auxin production rate is larger than the production rates of the other cells. Mathematically, we assume here that

$$\beta_a^i = \beta,\ \forall i \in V,\ \alpha_a^i = \alpha > 0,\ \forall i \neq i_0,$$

and that

$$\alpha_a^{i_0} \geq 2\sqrt{\beta/c} > \alpha. \tag{18}$$

The Instability criterion implies that, to be stable, the oriented graph $\mathcal{G}^*$ associated to $\boldsymbol{a}$ has to be composed of oriented trees pointing to roots. It contains thus at least one source. As regard of condition (10) of the main paper

$$(\alpha_a^i + \sum_{j\to i} \frac{1}{ca_j})^2 - 4\beta_a^i \frac{1}{c} \geq 0, \tag{19}$$

and (18), one gets that i_0 is the unique source and that the vein $\mathcal{G}^*$ must be a linear chain

$$i_0 \longrightarrow i_1 \longrightarrow i_2 \longrightarrow \cdots \longrightarrow i_n,$$

that is, must be a directed line with no vascular strands, which terminates in a sink i_n.

Consider the critical point $\boldsymbol{a}$ given by: $a_i = \frac{\alpha}{\beta}$ for $i \notin \{i_0, \dots, i_n\}$ and:

$$a_{i_0} = \frac{\alpha_a^{i_0} - \sqrt{(\alpha_a^{i_0})^2 - 4\beta_a \frac{1}{c}}}{2\beta_a},\ a_{i_k} = \frac{\alpha + \frac{1}{ca_{i_{k-1}}} - \sqrt{(\alpha + \frac{1}{ca_{i_{k-1}}})^2 - 4\beta_a^{i_k}\frac{1}{c}}}{2\beta} \text{ and } a_{i_n} = \frac{\alpha + \frac{1}{ca_{i_{n-1}}}}{\beta}$$

for $k = 1, \dots, n-1$.

Assume that $\beta > \frac{\mu}{2}$.

- When $\frac{-c\alpha^2+3\beta}{c\alpha^2-4\beta}\alpha + 2\sqrt{\frac{c^2\alpha^4\beta - 10c\alpha^2\beta^2 + 25\beta^3}{c(c\alpha^2-4\beta)^2}} \leq \alpha_0 \leq \frac{c\alpha^2+\beta}{c\alpha}$ and $c \leq \frac{\beta}{\alpha^2}$, the auxin flux increases along the vein, and takes values that are larger than the background auxin level α/β:

$$\frac{\alpha}{\beta} \leq a_{i_0} \leq a_{i_1} \leq \ldots \leq a_{i_{n-1}}, \tag{20}$$

with $n \leq L-1$ such that $a_{i_{n-1}} \leq \tilde{a}_c := \frac{2\sqrt{\beta/c}+\alpha}{4\beta-c\alpha^2}$. This parameter regime is illustrated in green in figure 9.

- if $\alpha_0 > \frac{c\alpha^2+\beta}{c\alpha}$ and $c \leq \frac{\beta}{\alpha^2}$ or $\alpha_0 > 2\sqrt{\frac{\beta}{c}}$ and $\frac{\beta}{\alpha^2} < c < 4\frac{\beta}{\alpha^2}$ then $n \leq L-1$ can be chosen arbitrarily and $\boldsymbol{a}$ has the property:
$$\frac{\alpha}{\beta} \geq a_{i_0} \geq a_{i_1} \geq \ldots \geq a_{i_{n-1}} \geq 0.$$

In both cases, $\boldsymbol{a}$ is locally asymptotically stable (by Theorem S.7.1).

References

C. Lobry, T. Sari, and S. Touhami. On tykhonovs theorem for convergence of solutions of slow and fast systems. *Electronic Journal of Differential Equations*, 1998(19):1–22, 1998.
